# Histone deacetylase inhibitors promote breast cancer metastasis by elevating NEDD9 expression

**DOI:** 10.1038/s41392-022-01221-6

**Published:** 2023-01-06

**Authors:** Zonglong Hu, Fan Wei, Yi Su, Yafang Wang, Yanyan Shen, Yanfen Fang, Jian Ding, Yi Chen

**Affiliations:** 1 Division of Anti-Tumor Pharmacology, Shanghai Institute of Materia Medica, Chinese Academy of Sciences, Shanghai, 201203 China; 2 University of Chinese Academy of Sciences, No.19A Yuquan Road, Beijing, 100049 China; 3 State Key Laboratory of Drug Research, Shanghai Institute of Materia Medica, Chinese Academy of Sciences, Shanghai, 201203 China

**Keywords:** Target identification, Drug development

## Abstract

Histone deacetylase (HDAC) is a kind of protease that modifies histone to regulate gene expression, and is usually abnormally activated in tumors. The approved pan-HDAC inhibitors have demonstrated clinical benefits for patients in some hematologic malignancies. Only limited therapeutic success in breast cancer has been observed in clinical trials. In this study, we declare that pan-HDAC inhibitors targeting NEDD9-FAK pathway exacerbate breast cancer metastasis in preclinical models, which may severely impede their clinical success. NEDD9 is not an oncogene, however, it has been demonstrated recently that there are high level or activity changes of NEDD9 in a variety of cancer, including leukemia, colon cancer, and breast cancer. Mechanistically, pan-HDAC inhibitors enhance H3K9 acetylation at the nedd9 gene promoter via inhibition of HDAC4 activity, thus increase NEDD9 expression, and then activate FAK phosphorylation. The realization that pan-HDAC inhibitors can alter the natural history of breast cancer by increasing invasion warrants clinical attention. In addition, although NEDD9 has been reported to have a hand in breast cancer metastasis, it has not received much attention, and no therapeutic strategies have been developed. Notably, we demonstrate that FAK inhibitors can reverse breast cancer metastasis induced by upregulation of NEDD9 via pan-HDAC inhibitors, which may offer a potential combination therapy for breast cancer.

## Introduction

Histone deacetylases (HDACs) deacetylate the histone lysine residues, remodel chromatin, and play an important role in gene transcription.^1^ Due to overexpression and aberrant activity in a variety of cancer subtypes, HDAC is considered a prospective and successful anticancer drug target, and already has been proved to be a successful target. Currently, several HDAC inhibitors have been approved by FDA for the treatment of hematological malignancies, including vorinostat (SAHA), belinostat (PXD-101), romidepsin (FK-228) and Panobinostat (LBH589).^2–4^ However, HDAC inhibitors have shown limited success in the treatment of solid tumors.^5^

Breast cancer is a highly heterogeneous disease in terms of histology, genetics and prognosis.^6,7^ It has been addressed that histone acetylation modification is one of the most easily intelligible epigenetic modifications, which has a key role in the development of breast cancer.^8^ Published studies demonstrate that HDAC inhibitors can inhibit breast cancer cells proliferation, influence mitosis and suppress DNA repair in the preclinical models, thereby producing anticancer effect.^9–11^ However, increasing evidence suggests that HDAC inhibitors show limited response in the clinical treatment of breast cancer. The present study attempts to confirm via preclinical models whether HDAC inhibitors have a potential clinical utility in breast cancer therapy.

*nedd9*, also named as *casl* and *hef1*, encodes a multi-domain scaffolding protein involved in cell signaling, and is known to regulate multiple cellular processes, such as cells proliferation, DNA damage response, migration, and so on.^12,13^ Although NEDD9 is not an oncogene, more and more literatures have reported that there are high level or activity changes of NEDD9 in leukemia, colon cancer, breast cancer, a variety of tumors.^12^ And multiple mouse models confirmed the carcinogenic role of NEDD9.^12^ A series of past studies have demonstrated that this protein represents an essential switch for prometastatic behaviors in multiple cancers, especially in triple negative breast cancer (TNBC).^12,14–17^ Some studies have confirmed that NEDD9 stimulates breast cancer cells invasion by influencing the epithelial-mesenchymal transition (EMT), and activating MMP.^14^ Loss of NEDD9 inactivates MMP14 and decreases the mobility of breast cancer cells.^14^ In oral squamous cell carcinoma, NEDD9 can increase MMP9 secretion and accelerate invadopodia formation.^18^ It has also been reported that the increased protein level of NEDD9 was significantly related to lymph node metastasis and tumor-node-metastasis stage, and indicated to a reduced 5-year survival of patients with TNBC.^15^ However, the regulatory mechanism of NEDD9 is still be uncertain. Recently, it has been reported that the abnormality of epigenetic modification is involved in affecting this gene expression, such as miR145-5p, miR-363-3p.^16,17^ And high level of miR-107 also has been proved to reduce NEDD9 level in breast cancer cells, and decreased cells migration and proliferation.^19^

In this study, we determined that pan-HDAC inhibitors elevate NEDD9 expression and promote breast cancer metastasis, which might be one of the reasons of its clinical failure. We also strive to clarify the mechanisms underlying pan-HDAC inhibitors in NEDD9 transcriptional activation, and attempt to overcome this effect by rational combination therapy.

## Results

### Pan-HDAC inhibitors promote breast cancer metastasis

**Fig. 1:**
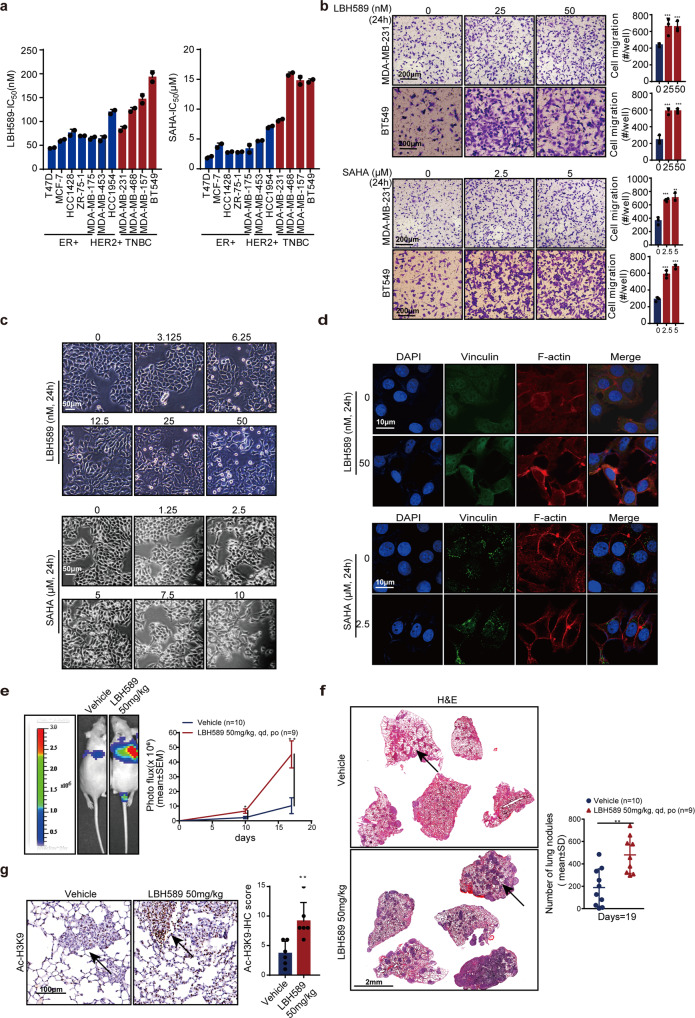
pan-HDAC inhibitors promote breast cancer metastasis. **a** Breast cancer cells treated with HDAC inhibitors LBH589 and SAHA, and the inhibitory effects on cell proliferation were measured using the sulforhodamine B (SRB) assay (mean IC_50_ values were calculated from at least three independent experiments). **b** HDAC inhibitors enhanced breast cancer cell invasiveness. Cells were treated with LBH589 or SAHA for 24 h at indicated doses, and were plated in transwell chambers for 12 h to evaluate the cell motility. Scale bar, 200 μm. **c** MCF-7 cell shape was observed after treatment with LBH589 or SAHA. Scale bar, 50 μm. **d** Immunofluorescence staining analysis of focal adhesion site number characterized by vinculin staining and actin-containing invadopodia in MCF-7 cells treated with HDAC inhibitors. Scale bar, 10 μm. **e**–**g** The stimulation of breast tumor metastasis in vivo after treatment with LBH589 was assessed. Representative images and quantitative analysis of lung metastatic nodules of BalB/c female nude mice detected by in vivo luciferase-based bioluminescence imaging in **e**, H&E-stained lung sections and quantification of lung metastatic nodules of mice in **f**, scale bar, 2 mm. Representative images on Ac-H3K9-stained lung sections and quantification of Ac-H3K9 expression in **g**, scale bar, 100 μm. The arrows indicate Ac-H3K9 staining. The results are expressed as mean ± SD. Two-tailed Student’s *t*-tests were performed. *p < 0.05, **p < 0.01, ***p < 0.001

We first detected the effects of HDAC inhibitors on the proliferation and motility of breast cancer cells. We used SAHA and LBH589, two of the first FDA-approved HDAC inhibitors. As expected, these two HDAC inhibitors suppressed breast cancer cell proliferation independent of breast tumor subtype, with IC_50_ values ranging from 1 µM to 20 µM for SAHA and 30–200 nM for LBH589 (Fig. 1a and Supplementary Fig. 1a). To our surprise, both SAHA and LBH589 stimulated breast cancer cell migration and invasion at cellular level in vitro (Fig. 1b), although it has been pointed out that LBH589 inhibited metastasis of triple-negative breast cancer.^20^ Transwell invasion assays demonstrated that 25 nM LBH589 stimulated a nearly two-fold increase in the invasiveness of MDA-MB-231. Changes in cell morphology were also observed following SAHA or LBH589 treatment. Compared with control, MCF-7 cells showed loss of cell-cell adhesion, instead of a cobblestone morphology in monolayer culture, displaying a discernible fibroblast-like morphology after treatment with LBH589 or SAHA (Fig. 1c). Furthermore, immunofluorescence staining showed a marked increase in vinculin-containing focal adhesion sites, and actin-containing invadopodia in HDAC inhibitors-treated MCF-7 and MDA-MB-231 cells, which are collectively required for cell invasion (Fig. 1d and Supplementary Fig. 1b). Vinculin usually interacts with integrins, regulate focal adhesion structure and functions, and then regulates cell shape.^21^

We next employed an experimental metastasis model to query whether HDAC inhibitors stimulate metastasis in vivo. The luciferase labeled breast cancer cell MDA-MB-231/4175 were inoculated to nude mice via tail vein injection, and the metastatic colonization of lung was evaluated. Consistent with in vitro results, nude mice treated with 50 mg/kg LBH589 displayed a significantly higher number of lung metastases relative to mice treated with the vehicle (Fig. 1e, f). This increase was accompanied by increased acetylation of H3K9 (Ac-H3K9) within the lung (Fig. 1g). These data indicate that pan-HDAC inhibitors could stimulate breast cancer metastasis.

### HDAC inhibitors increase NEDD9 expression in breast cancer

**Fig. 2:**
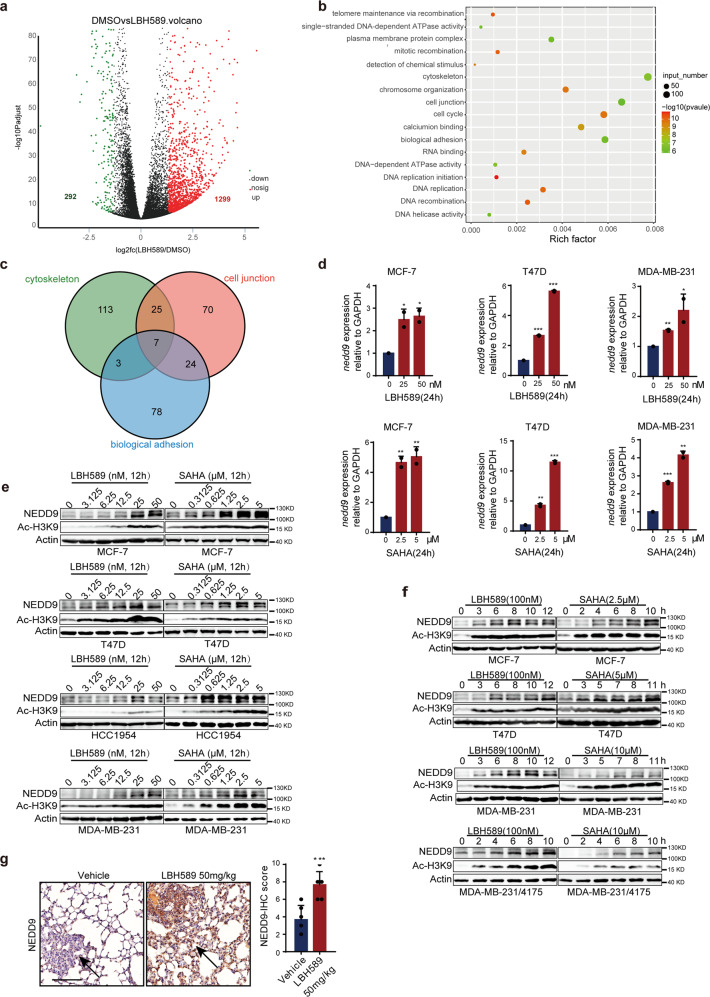
HDAC inhibitors upregulate NEDD9 expression in breast cancer cells. **a** RNA sequencing analysis in MCF-7 cells treated with LBH589. 1299 genes were upregulated (p_adjust < 0.001, fold change > 2.5) and 292 genes were downregulated (p_adjust < 0.001, fold change < 0.4) in MCF-7 cells after 50 nM LBH589 treatment. **b** GO enrichment analysis. 146 GO of different biological functions were enriched by analyzing 1591 significantly changed genes in **a**. **c** Venn analysis. Seven genes were obtained by intersected cytoskeletal, cell junction and biological adhesion associated with cell migration in **b**. Relative *nedd9* mRNA (**d**) and protein levels (**e**, **f**) induced by the HDAC inhibitors LBH589 and SAHA. **g** IHC staining indicated that NEDD9 expression was upregulated in lung metastatic nodules of lung sections after administrating 50 mg/kg LBH589. Scale bar, 100 μm. The arrows show NEDD9 staining. Wald test is used to analyze the differential genes expression and the Fisher ‘ s exact test is employed to GO enrichment analysis. Other results are expressed as mean ± SD. Two-tailed Student’s *t*-tests were performed. *p < 0.05, **p < 0.01, ***p < 0.001

Considering that HDAC inhibitors affect gene transcription, we analyzed gene changes in LBH589-treated MCF-7 cells using RNA-seq. LBH589 treatment led to differential expression of 1591 genes (p_adjust <0.001, fold change >2.5 and fold change <0.4), including 292 downregulated and 1299 upregulated genes (Fig. 2a, Supplementary Fig. 2a and Supplementary Table. 1). GO pathway enrichment analysis was performed, and 146 genes were found to be significantly altered (Fig. 2b and Supplementary Table 2). And most of the changes were focus on cell junction, adhesion, and cytoskeletal signal pathways. Further, seven genes were obtained by intersected analysis with Venn (Fig. 2c and Supplementary Table 3). NEDD9, which has been identified as a marker of metastasis in the past decade, is one of the most significantly upregulated transcripts of LBH589.^12^ Consistent with the RNA-seq results, compared with the control, HDAC inhibitors induced a notable upregulation of NEDD9 mRNA (Fig. 2d) and protein (Fig. 2e, f and Supplementary Fig. 2b). Maximal increases were achieved 12 h after treatment with SAHA 5 μM or LBH589 50 nM, accompanied by increasing Ac-H3K9. Immunohistochemistry (IHC) staining indicated that NEDD9 was upregulated in mice lung metastatic nodules after treatment with 50 mg/kg LBH589 (Fig. 2g). Taken together, the results suggest that SAHA and LBH589 increase NEDD9 mRNA and protein expression in breast cancer cells.

### NEDD9 is an operative target in HDAC inhibitor-promoted breast cancer metastasis

**Fig. 3:**
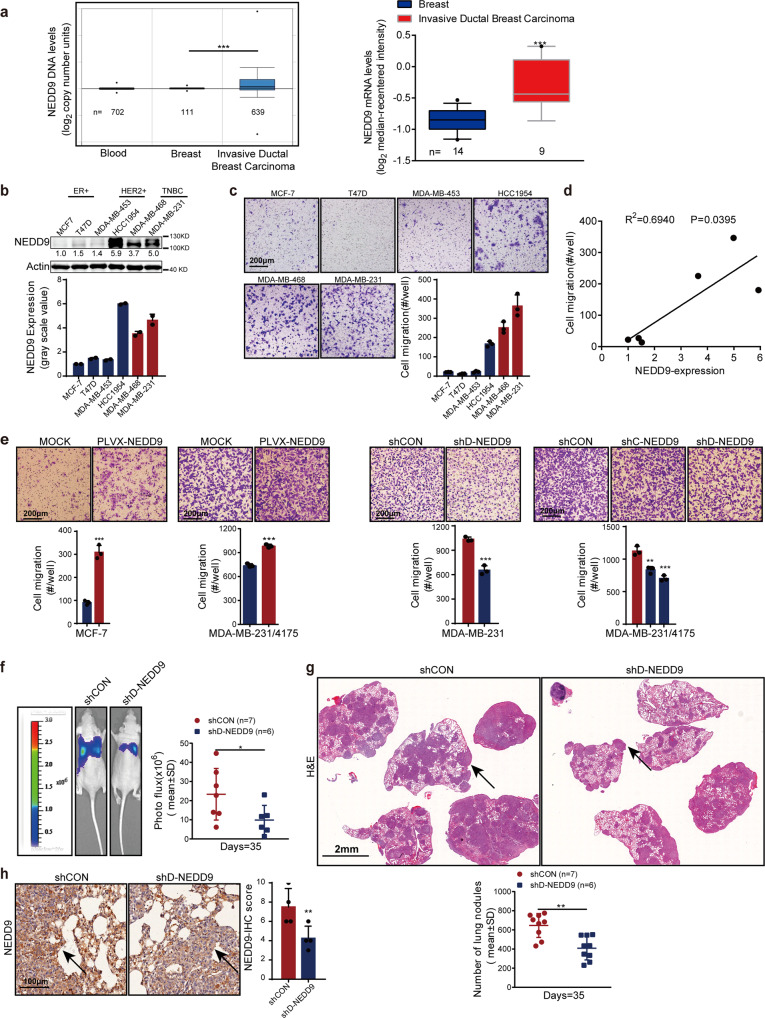
NEDD9 promotes breast cancer metastasis. **a** Cancer Genome Atlas (TCGA) Breast cohort showed that the copy numbers of NEDD9 DNA in invasive ductal breast carcinoma are higher than that in normal breast tissues. Ma Breast 4 cohort indicated that NEDD9 mRNA levels were also significantly higher in invasive ductal breast carcinoma patients. NEDD9 expression (**b**) and mobility (**c**) were evaluated in a panel of breast cancer cell lines. Scale bar, 200 μm. **d** NEDD9 expression levels in breast cancer cell lines were quantified by Image J, and the correlation between NEDD9 and cells motility was analyzed using GraphPad. **e** Cells motility in NEDD9 overexpressed and knockdown cells were evaluated. Scale bar, 200 μm. The formation of lung metastatic nodules following MDA-MB-231/4175 cells treated with NEDD9 siRNA were detected in vivo by luciferase-based bioluminescence imaging (**f**), H&E-stained lung sections and quantified (**g**). Scale bar, 2 mm. **h** Representative images on NEDD9 expression in lung sections. Scale bar, 100 μm. The arrows indicate NEDD9 staining. The results are expressed as mean ± SD. Two-tailed Student’s *t*-tests were performed. *p < 0.05, **p < 0.01, ***p < 0.001

Although in some published paper, NEDD9 is a pro-metastasis gene in melanoma and triple-negative breast cancer, the results are not always consistent.^22,23^ We confirmed the function of NEDD9 in breast cancer metastasis. Analysis of paired samples in the Cancer Genome Atlas (TCGA) breast cohort showed a significant upregulation of the NEDD9 DNA copy number in invasive ductal carcinoma relative to normal breast tissues (Fig. 3a). Similar results were observed in oncomine database of the Ma Breast 4 cohort Statisties.^24^ Compared with normal breast tissues, in invasive ductal carcinoma tissues showed a high level of NEDD9 mRNA (Fig. 3a). Moreover, we attempted to determine whether the high level of NEDD9 correlated with the high mobility in human breast cancer cells. NEDD9 levels were determined in a small panel of human breast cancer cell lines (Fig. 3b). Mobility of these cells was determined by invasion and wound healing assays at the same time (Fig. 3c and Supplementary Fig. 3a). The results demonstrated a notable correlation between mobility and the level of NEDD9 (Fig. 3d). Further, compared with the parent cells, cells that overexpress NEDD9 promoted MCF-7 and MDA-MB-231 cell invasion in vitro (Fig. 3e). In contrast, infection with different NEDD9 shRNAs significantly reduced NEDD9 in MDA-MB-231 and MDA-MB-231/4175 cells, while simultaneously suppressing cell invasion (Fig. 3e). However, overexpression and knockdown of NEDD9 have limited effects on cell proliferation (Supplementary Fig. 3b, c). In accord with our in vitro results, NEDD9 depletion in MDA-MB-231/4175 cells reduced lung metastatic nodule formation in experimental lung metastasis model relative to shCON cells (Fig. 3f, g). Meanwhile, IHC staining confirmed that the level of NEDD9 was reduced in shD-NEDD9 tumor tissue (Fig. 3h). Our results suggest that NEDD9 is essential for the promotion of breast cancer metastasis.

**Fig. 4:**
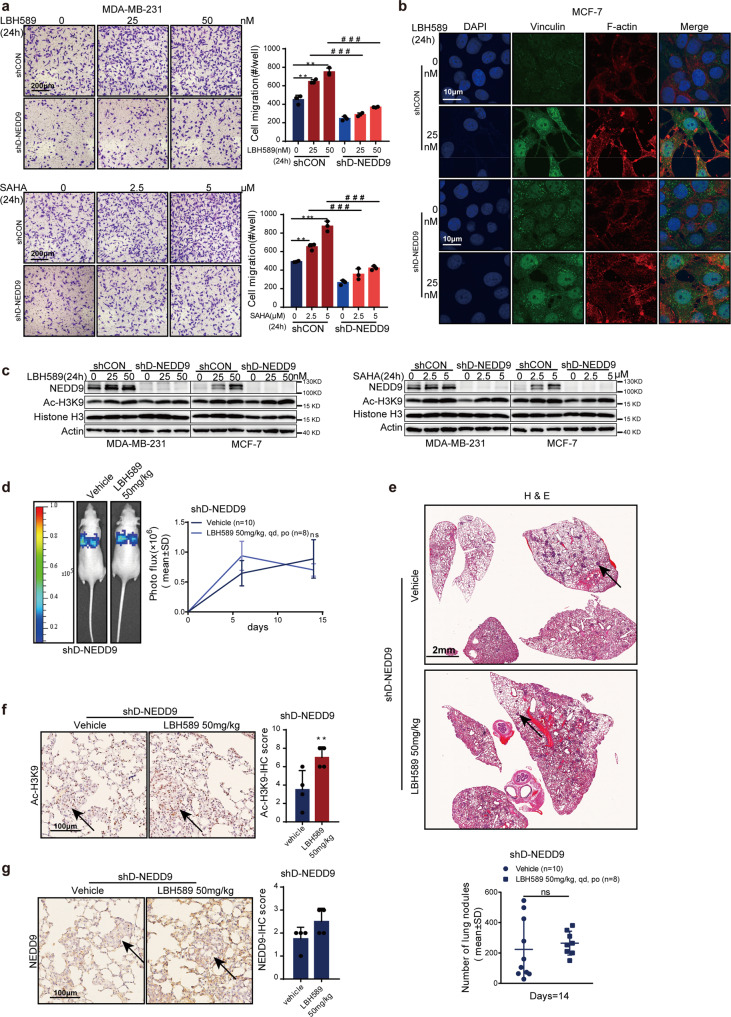
NEDD9 loss reverses HDAC inhibitors-promoted metastasis. **a** Invasiveness was reversed when NEDD9 was depleted in HDAC inhibitors treated cells. Scale bar, 200 μm. **b** Immunofluorescence staining analysis of focal adhesion sites number characterized by vinculin staining and actin-containing invadopodia in MCF-7 and MCF-7 sh NEDD9 cells treated with HDAC inhibitors. Scale bar, 10 μm. **c** Western blotting analysis showed that knockdown of NEDD9 could impair the increase in NEDD9 expression induced by HDAC inhibitors SAHA and LBH589 in a dose-dependent manner in MDA-MB-231 and MCF-7 cells. The in vivo therapeutic effect of LBH589 on NEDD9- depleted MDA-MB-231/4175 cells metastasis was evaluated by in vivo luciferase-based bioluminescence (**d**) and H&E (**e**). Immunohistochemical staining analysis of Ac-H3K9 (**f**) and NEDD9 (**g**) in lung tissues from each group. Scale bar, 100 μm. The arrows indicate positive staining. Representative sections are shown. Results are expressed as mean ± SD. Two-tailed Student’s *t*-tests were performed. *p < 0.05, **p < 0.01, ***p < 0.001. ns not significant

If the stimulating effect of HDAC inhibitors on NEDD9 is vital to promote tumor cells’ invasion ability, we would expect that loss of NEDD9 could reverse HDAC inhibitors-induced phenotypes. Therefore, MDA-MB-231 cells with low NEDD9 expression were treated with HDAC inhibitors again. As expected, knockdown of NEDD9 using shRNAs partially restored HDAC inhibitor-promoted MDA-MB-231 invasion in vitro (Fig. 4a). Moreover, immunofluorescence staining indicated that inhibition of NEDD9 could also suppress vinculin-containing focal adhesion number and actin-containing invadopodia in LBH589-treated MCF-7 cells (Fig. 4b). Western blotting confirmed that HDAC inhibitors LBH589 and SAHA could not increase NEDD9 expression in shD-NEDD9 MDA-MB-231 and MCF-7 cells (Fig. 4c). Notably, no marked increase in nodule formation in lung after treatment with LBH589 50 mg/kg was found in mice that received NEDD9-depleted MDA-MB-231/4175 cells (Fig. 4d, e). H3K9 acetylation increased (Fig. 4f), within lung nodules. These data support the theory that increased NEDD9 expression causes the increased invasiveness of breast cancer cells after HDAC inhibitors treatment.

### NEDD9 phosphorylates FAK and reveals new therapeutic opportunities

**Fig. 5:**
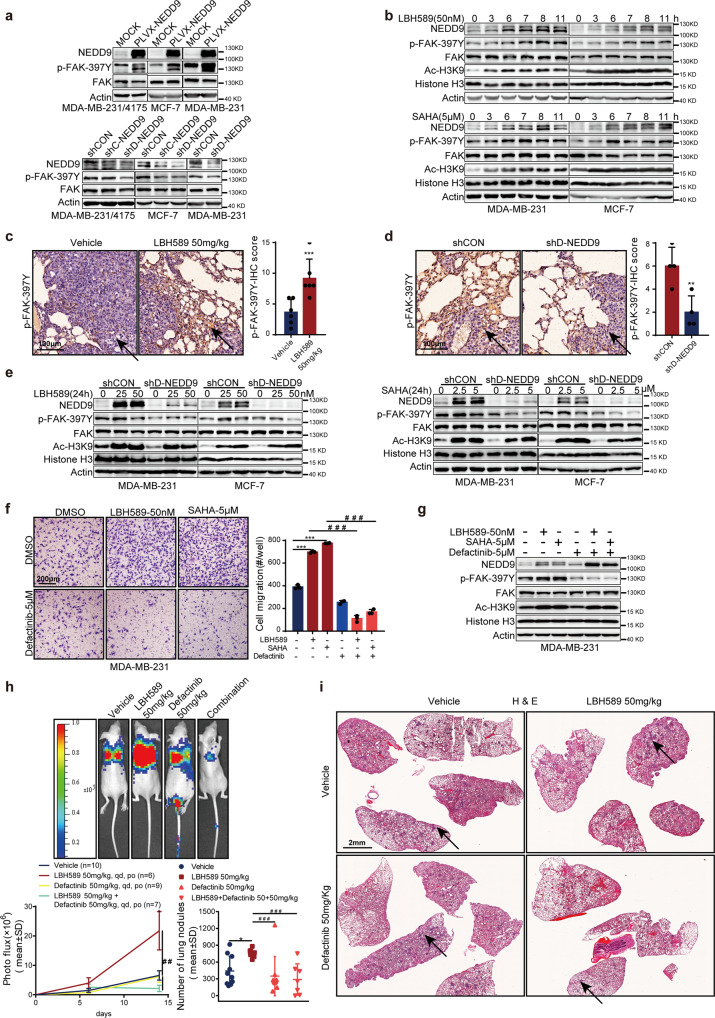
Phosphorylated FAK is the potential downstream of NEDD9 regulated by HDAC inhibitors. Phosphorylated FAK (p-FAK-397Y) level in breast cancer cells transfected with NEDD9-shRNA (**a**, upper panel), PLVX-NEDD9 (**a**, lower panel), or treated with HDAC inhibitors (**b**). Representative images showing IHC staining of phosphorylated FAK in metastasis from mice treated with LBH589 (**c**) and from stable NEDD9-knockdown models (**d**). Scale bar, 100 μm. The arrows show positive staining. **e** p-FAK-397Y expression was analyzed in NEDD9-depleted breast cells after treatment with HDAC inhibitors. FAK inhibitor defactinib reversed HDAC inhibitors-mediated breast cancer cells invasion (**f**), Scale bar, 200 μm, FAK phosphorylation (**g**), and lung metastasis in vivo (**h**, **i**). The in vivo therapeutic effect of defactinib and LBH589 combination on lung metastasis was evaluated by bioluminescence (**h**) and histological examinations (**i**). Scale bar, 2 mm. The results are expressed as mean ± SD. Two-tailed Student’s *t*-tests unless noted. */#*p* < 0.05, **/##*p* < 0.01, ***/###*p* < 0.001

As previous studies have identified, NEDD9 is known for its role in increasing phosphorylation of FAK at Tyr397 (p-FAK-397Y) to promote cancer metastasis.^23,25^ We next detected whether p-FAK-397Y was increased by HDAC inhibitors. As shown in Fig. 5a, the increased NEDD9 stimulated FAK phosphorylation, while depletion of NEDD9 led to a decreased p-FAK-397Y. Furthermore, we also determined that HDAC inhibitors time-dependently increased p-FAK-397Y expression, while also increasing NEDD9 expression in MCF-7 and MDA-MB-231 cells (Fig. 5b). The expression level of p-FAK-397Y was also upregulated in 50 mg/kg LBH589 treated mice group compared with vehicle mice (Fig. 5c), and downregulated in shD-NEDD9 tumor tissue (Fig. 5d), as determined by IHC. Similarly, depletion of NEDD9 blunted the upregulation of p-FAK-397Y by HDAC inhibitors (Fig. 5e and Supplementary Fig. 4a). As expected, the FAK inhibitor defactinib (5 μM) inhibited FAK phosphorylation and restrained cell invasion in the presence of HDAC inhibitors (Fig. 5f, g). Consistent with our in vitro data, we also observed that 50 mg/kg defactinib completely reversed LBH589-induced lung metastasis in the MDA-MB-231/4175 animal model (Fig. 5h, i). IHC staining demonstrated that H3K9 acetylation, p-FAK-397Y, and NEDD9 levels were altered within lung nodules (Supplementary Fig. 4b, c and d). And there was no reduction in body weight after treatment with LBH589, defactinib alone and both (Supplementary Fig. 4e). These results indicate that HDAC inhibitors promote breast cancer metastasis by NEDD9-induced phosphorylation of FAK. FAK inhibitor-mediated reversal of HDAC inhibitor-mediated breast cancer metastasis suggests the combinatorial therapeutic opportunities of FAK and HDAC inhibitors in breast cancer.

### HDAC4 inhibition increases histone acetylation at NEDD9 promoter and promotes NEDD9 transcription

**Fig. 6:**
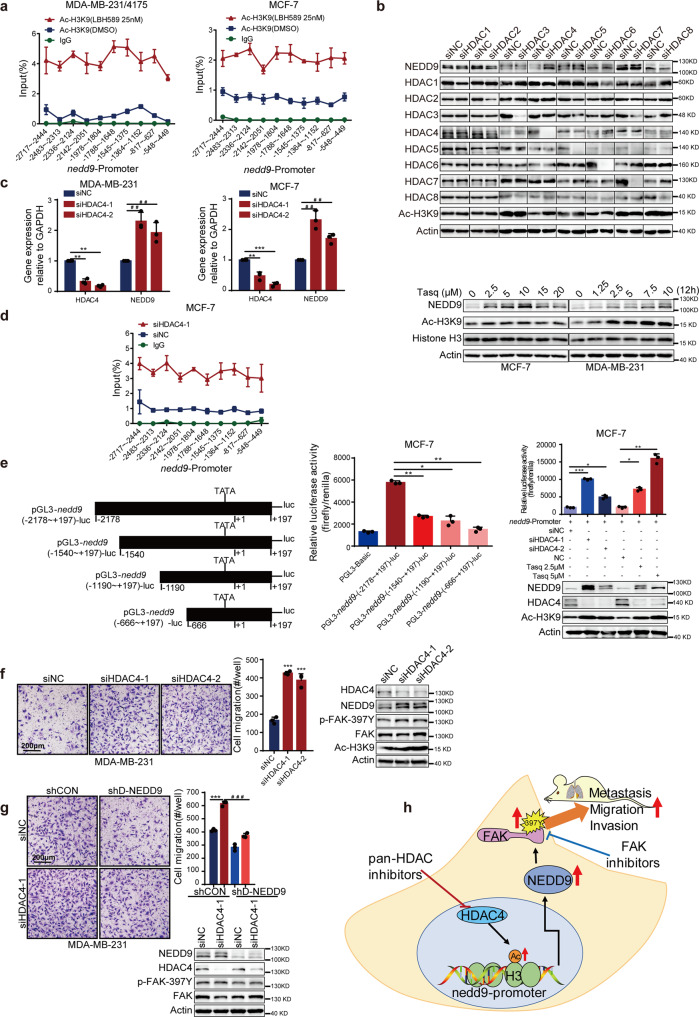
HDAC4 inhibition upregulates NEDD9 expression and promotes breast cancer cells migration. **a** ChIP assay of recruitment of Ac-H3K9 to the *nedd9* promoter region was observed after cells treatment with LBH589. **b** Western blotting analysis of NEDD9 expression after cells transfected with indicated siRNA or treated with HDAC4 specific inhibitor tasquinimod. MCF-7 and MDA-MB-231 cells were transfected with two independent HDAC4 siRNAs, then NEDD9 mRNA levels (**c**), *nedd9* promoter ChIP assay (**d**), *nedd9* promoter luciferase activity (**e**) and cells migration ability (**f**) were examined. Scale bar, 200 μm. **g** Invasion ability was restored when HDAC4 was depleted by siRNA in NEDD9 knockdown cells. Scale bar, 200 μm. **h** Schematic diagram depicting the regulation of NEDD9 in breast tumor cells by HDAC4 inhibition contributes to breast metastasis. Results are expressed as mean ± SD. Two-tailed Student’s *t*-tests were performed. */#*p* < 0.05, **/##*p* < 0.01, ***/###*p* < 0.001

HDAC inhibitors are thought to induce gene expression via alterations in acetylation modification of histone, leading to induction of gene expression. To further understand how HDAC inhibitors regulate NEDD9, ChIP analysis was applied to observe the transcriptional regulation of NEDD9 mediated by HDACs. Data shown in Fig. 6a and Supplementary Fig. 5a revealed that HDAC inhibitors could significantly stimulate H3K9ac recruitment to the promoter of the *nedd9* in MCF-7, T47D and MDA-MB-231 cells.

Given that HDAC family members regulate different biological pathways and have different functions, we next attempted to illustrate whether specific HDAC family member was responsible for upregulation of NEDD9. MCF-7 and MDA-MB-231 cells were transfected with siRNAs to knock down HDACs (HDAC1, 2, 3 and 8 for class I, and HDAC4, 5, 6 and 7 for class II) individually. Among which, only HDAC4 silencing led to increased NEDD9 mRNA and protein expression (Fig. 6b, c). Simultaneously, the HDAC4-specific inhibitor tasquinimod also upregulated the protein level of NEDD9 (Fig. 6b). Consistent with previous results, we observed increased levels of H3K9 acetylation in the *nedd9* promoter when HDAC4 was depleted (Fig. 6d). Then promoter activity was measured using a pGL3-*nedd9*-luc reporter system to further confirm that HDAC4 regulates *nedd9* transcription. According to our ChIP assay results, four different *nedd9* promoter fragments were transformed into pGL3 basic luciferase reporter vectors (Fig. 6e). As expected, *nedd9* transcription was stimulated by inhibition of HDAC4 with siRNA or tasquinimod in MCF-7 cells, using the pGL3-NEDD9 -luc system (Fig. 6e). Transwell assays were used to detect the mobility of HDAC4-silenced cells. As expected, loss of HDAC4 facilitated breast cancer cell invasion in vitro (Fig. 6f). No marked increase in p-FAK-397Y was observed when silencing HDAC4 in NEDD9 deficient MDA-MB-231 cell (Fig. 6g). Knockdown of HDAC4 did not have significant effect on cell proliferation (Supplementary Fig. 5b). Taken together, the findings support the theory that increased NEDD9 expression induced by HDAC4 inhibition promotes breast cancer cell mobility.

## Discussion

The approval of SAHA in 2006 for cutaneous T-cell lymphoma demonstrates that HDAC is a potential cancer therapeutic target, and that small molecule drugs targeting HDAC might be promising in a wide range of cancer types. Existing evidences indicate that HDAC inhibitors repress breast cancer cells proliferation,^26,27^ angiogenesis,^28^ and regulate anti-tumor immunity.^29,30^ Furthermore, several studies have demonstrated that HDAC regulates events implicated in breast cancer progression, including self-renewal and expansion of stem cells, invasion and metastasis.^31^ Numerous clinical trials are underway to evaluate the use of HDAC inhibitors against breast cancer. However, the efficacy of HDAC inhibitors as monotherapy in breast cancer remain disappointing. A California cancer consortium study revealed that SAHA alone failed to achieve success in the treatment of 14 patients with metastatic breast cancer.^32^ In our study, we observed that the pan-HDAC inhibitors SAHA and LBH589 promote breast cancer invasion and metastasis in vitro and in vivo, which in turn will impede the efficacy of HDAC inhibitors in breast cancer. Previous studies have shown evidence that HDAC inhibitors exert potent anti-metastatic activity.^20,33–35^ However, recently investigations demonstrated that HDAC inhibitors promote metastasis of hepatoma, melanoma, and colorectal cancer cells.^36–38^ Here, we determined that SAHA and LBH589 changed the shape of breast cancer cells to a fibroblast-like morphology, increased cells invasiveness, and promoted lung metastasis in vivo. All these results strongly suggest that HDAC inhibitors as monotherapy in breast cancer should be avoided.

Furthermore, we revealed a novel mechanism by which HDAC inhibitors regulate tumor metastasis. Previously reported mechanisms include snail-^37,38^ and N-cadherin-induced EMT.^36^ In this study, we observed for the first time that pan-HDAC inhibitors increase the expression of NEDD9 and trigger metastasis. Over nearly two decades ago, altered expression of the NEDD9 was identified as a contributing factor to cancer metastasis in many different kinds of cancer. However, although studies have identified elevated NEDD9 expression as prometastatic previously, other studies have suggested an anti-metastatic role in breast cancer metastasis.^22,39^ We demonstrated that NEDD9 overexpression promoted invasiveness of breast cancer cells. In contrast, depletion of NEDD9 using siRNA inhibited the invasive phenotype of breast cancer cells in vitro and in vivo. HDAC4 inhibition increased histone acetylation at the *nedd9* gene promoter. Consistent with previous reports,^23,25^ we demonstrated that upregulated NEDD9 activates FAK phosphorylation and contributes to HDAC inhibitor-induced metastasis. As a scaffolding protein, NEDD9 contains multiple protein–protein interaction domains for docking with partner proteins. It is reasonable to presume that NEDD9 provides a binding site for FAK, increases FAK phosphorylation, and regulates cell migration. However, it also has been reported that there is a positive feedback loop between NEDD9 and FAK.^40^ All these will be studied in our future research. Beyond these mechanistic insights, this study attempted to explore a mechanism-based rationale for combination therapy to overcome HDAC inhibitor side effects. Owing to the lack of a kinase domain, NEDD9 represents a difficult molecule to target.^41^ As a result, we focused our attention on indirect NEDD9 inhibition via FAK. FAK is an emerging target in cancer therapies, due to its probable role in tumor metastasis.^42^ However, FAK inhibitors only have modest clinical activity in the clinical trials, and now many efforts are focused on exploring efficient combinational therapies.^41^ As expected, concurrent inhibition of FAK by FAK specific inhibitor defactinib reversed HDAC inhibitor-induced breast cancer metastasis. Therefore, it is worth trying to evaluate the benefit of this combinatorial approach in clinical trials to improve the efficacy of HDAC inhibitors and FAK inhibitors in breast cancer therapy.

It is well established that HDACs are critical regulators of gene expression owing to enzymatic removal of the acetyl group from histones or non-histones.^31^ Scientists have identified nearly 18 HDAC isoforms in humans,^43^ and grouped into five classes. HDACs are known to modulate diverse physiological and pathological activities.^1^ The biological effect of HDAC inhibition is dependent on drug specificity, and intrinsic operation of cell-signaling pathways. Several related studies have revealed aberrant expression of different HDAC isoforms in various types of tumors.^44–48^ The overexpression of individual isoforms serve as biomarkers in different tumors implicating its initiation and progression. Thus, we investigated which HDAC isoforms had any effect on NEDD9 expression. We found a limited relationship between NEDD9 and HDAC isoforms, with the exception for HDAC4. The most important observation in the present study is the provision of a detailed mechanism of HDAC4 inhibition induced-metastasis. We identified HDAC4 inhibition as a positive regulator of NEDD9 expression via elevating acetylation of the *nedd9* promoter during breast cancer cell invasion and metastasis. However, we also found that SAHA could increase NEDD9 in HUVECs, which might improve the mobility of HUVECs and be involved in HDAC inhibitors-induced metastasis (Supplementary Fig. 2c). All these results suggest that pan-HDAC inhibitors with HDAC4 inhibitory activity are capable of producing deleterious effects on breast cancer progression. New strategy involving HDAC inhibitor development is that identify more potent, isoform specific inhibitors.

In conclusion, we demonstrated that inhibition of HDAC4 stimulated NEDD9-FAK signaling pathway, leading to metastasis of breast cancer cell, and consequently limiting the clinical efficacy of pan-HDAC inhibitors against breast cancer (Fig. 6h). Our results emphasize the importance for selective HDAC inhibitors research and development. Notably, FAK inhibitors were found to reverse breast metastases induced by non-specific HDAC inhibitors, although further evidence is needed.

## Materials and methods

### Cell lines

CAL-51 was got from DSMZ (Germany). BT-549 was acquied from the cell bank of the Chinese Academy of Sciences (Shanghai, China). Professor Joyce Slingerland (University of Miami) generously provided MDA-MB-231/4175. The remaining breast cancer cell lines were obtained from the ATCC (Manassas, VA, US). All cells used in this study were cultured as suggested and identified by STR sequence analysis.

### Reagents and antibodies

SAHA (#S1047), LBH589 (#S1089), Defactinib (#S7654) and Tasquinimod (#7617) were received from Selleck (Texas, USA). Antibodies NEDD9 (#4044), phosphor-Src-Tyr416 (#6943), Src (#2108 S), phosphor-FAK-Tyr397 (#8556 S), FAK (#3285 S), HDAC1 (#34589 T), HDAC2 (#5113 T), HDAC3(#3949 S), HDAC4 (#5392 S), HDAC5 (#20458 S), HDAC6 (#7558 S) were purchased from Cell Signaling Technology (Massachusetts, USA). Ac-H3K9 (#ab10812) was purchased from Abcam (Cambridge, UK). vinculin(#66305-1-Ig), Histone H3 (17168-1-AP), Actin (#60008-1-Ig), were purchased from Proteintech (Chicogo, USA).

### Plasmids and transfection

The PLKO.1-shNEDD9 plasmids and PLVX-NEDD9 plasmid were acquired from Generay Biotech Co., Ltd. (Shanghai, China). Lentivirus packing plasmid psPAX2 and envelope plasmid pMD2.G were obtained from Addgene. Transfection was performed by Lipofectamine 2000 (Invitrogen, CA, US). MCF-7, MDA-MB-231, and MDA-MB-231/4175 were infected with lentivirus in the presence of polybrene (Sigma-Aldrich, St Louis, MO, US) and positively screening with puromycin (Sigma-Aldrich).

### Transwell assay

Cell invasion was measured using 8-μm-pore 24-well transwell plates (Corning, New York, US, #3422). The upper layer was inoculated with serum-free cells, and placed in medium with 10% serum. After 12 h of culture, cells were fixed with 90% ethanol, wiped dry, and dyed with 1% crystal violet. Migrated cells were counted by selecting three fields per filter (20× magnification).

### Immunofluorescence

MCF-7 cells grown for 24 h on glass coverslips (10^5^ cells per well 12-well plates). The coverslips were cultured with the primary antibody vinculin (1:100, #66305-1-Ig, Proteintech, Chicogo, US) overnight. Then the secondary antibody Alexa Fluor 488 goat anti-mouse IgG (H + L) (1:100, #33206ES60, Yeasen, Shanghai, China) was used to incubate the coverslips for 1 h after PBS wash. Nuclei were indicated with 4,6-diamidino-2-phenylindole (1:50, #40727ES10, Yeasen). TRITC phalloidin (1:100, #40734ES75, Yeasen) for F-actin staining was contained during secondary antibody incubation. Cells were photographed and analyzed using Olympus FluoView 1000.

### RNA purification and RT-PCR analysis

According to the manual, mRNA was extracted with Trizol and reverse-transcribed using RT SuperMix kit for qPCR (#R233-01 Vazyme, Nanjing, China). SYBR Green PCR master mix was applied to perform RT-qPCR (#6252, Biorad, California, US). And 2^-ΔΔCT^ method was used to quantify relative gene expression. Primers sequences are as follows: NEDD9: ATGGCAAGGGCCTTATATGACA and TTCTGCTCTATGACGGTCAGG;

HDAC4: GGCCCACCGGAATCTGAAC and GAACTCTGGTCAAGGGAACTG;

GAPDH: GGAGCGAGATCCCTCCAAAAT and GGCTGTTGTCATACTTCTCATGG.

### Immunoblotting analysis

Cells were lysed in 2% SDS buffer (, #A100227, Sangon Biotech), quantified by BCA assay (#23225, Thermo Scientific, Massachusetts, US), and incubated with the indicated antibodies: NEDD9 (#4044, Cell signaling Technology (CST), Massachusetts, US); phospho-FAK (Tyr397) (p-FAK-397Y) (#8556 S, CST); acetyl-histone H3 (Lys9) (Ac-H3K9) (#ab10812, Abcam, Cambridge, UK); histone-H3 (17168-1-AP, Proteintech); FAK (#3285 S, CST); phospho-Src (Tyr416) (p-416Y-Src) (#6943, CST); Src (#2108 S, CST); HDAC1 (#34589 T, CST); HDAC2 (#5113 T, CST); HDAC3 (#3949 S, CST); HDAC4 (#5392 S, CST); HDAC6 (#7558 S, CST); Actin (Proteintech).

### siRNA transfection

siRNAs ordered from GenePharma (Shanghai, China) were transfected with Lipofectamine RNAi MAX Reagent (#13778150, Invitrogen) for 48 h. The sequences were as follows:

siHDAC1: CGGUCAUGUCCAAAGUAAUTT; siHDAC2: UGUGAAGUUAAACCGACAATT; siHDAC3: AAUCAGAACUCACGCCAGUTT; siHDAC4-1: CGAGCACUGUGGUUUACAATT; siHDAC4-2: GGAAUCUGAACCACUGCAUTT; siHDAC6:GCAAUGGAAGAAGACCUAATT; siHDAC5: CGGGUUUGAUGCUGUUGAATT; siHDAC7: GGAGGAAGAACCUAUGAAUTT; siHDAC8: GGUCCCGGUUUAUAUCUAUTT.

### Animal studies

All animal experiments were conducted in accordance with the institutional ethical guidelines on laboratory animal care, with the approval of the Institute of Animal Care and Use Committee at the Shanghai Institute of Materia Medica (No. 2017-04-DJ-26, 2018-05-DJ-37). Female BALB/c nude mice (4–5 weeks, 16–20 g) were maintained under pathogen-free conditions with sufficient food and water. Mice were allocated to experimental groups using random number tables. Luciferase-labeled MDA-MB-231/4175 cells were injected systemically via the tail vein (10^6^ cells/per mouse) to generate a metastatic model to mimic breast cancer lung metastasis. LBH589 (0.9% NaCl + ddH_2_O) and defactinib (5% DMSO + 50% PEG300 + 5%Tween 80) were given orally daily. Evidence of lung metastasis was determined in real time by luciferase-based noninvasive bioluminescence imaging using the IVIS Lumina II (PerkinElmer, Massachusetts, US). Alternatively, lung tissues were surgically removed, and the number of lung metastasis nodules were counted and examined by H&E staining.

### Immunohistochemistry (IHC)

Lung tissues were fixed in 4% paraformaldehyde, dehydrated, paraffin-embedded, and deparaffinized before being incubated with NEDD9 antibody (1:200, #ab18056, Abcam), Anti-Histone H3 (acetyl K9) (1:200, #ab10812, Abcam) and Phospho-FAK (Tyr397) (1:200, # 44-624 G, Invitrogen) at 4 °C overnight. Secondary antibodies were then incubated at 37 °C for 30 min.

IHC was independently assessed by pathologists from ZuoCheng Biological Technology LTD, Shanghai, China. They assessed the expression of proteins using the quick score method, which considers both the proportion and intensity of stained cells. Briefly, the proportion of positively stained cells was measured on a scale of 1–6 (1:1–4%; 2: 5–19%; 3: 20–39%; 4: 40–59%; 5: 60–79%; and 6: 80–100%). The staining intensity of positive cells was scored from no staining 0 to strong staining 3. The immunohistochemistry score was calculated by multiplying the percentage score by the intensity score, ranging from value of 0 to 18.

### ChIP assay

SimpleChIP Plus Enzymatic Chromatin IP Kit (#9005, CST) was used for ChIP assasy. Antibodies used were as follows: anti-IgG (1:100, #2729, CST), and anti-acetyl-histone H3 (Lys9, H3K9ac, 1:100, #ab10812, Abcam). Extracted DNA was used as a template in qPCR reactions, with primers encompassing *nedd9* promoter (–3000~–400). The sequences of primers are as follows: –2717~–2444:GTATGCGATAGTTAATGAATGGG and GTGTCTATTTATGATTCCATTTGCTT; –2483~–2313: GAGGGGAATGAGTCAAGCAAATG and GCTGTTGTATGGAGTGGAGGATG; –2336~–2124: TCATCCTCCACTCCATACAACAG and AGGACAGGGCTTACATTTTCATC; –2142~–2051: AAAATGTAAGCCCTGTCCTCAAT and CAACATTTTGGCAAACCACAGAT; –1978~–1804:GTAGGAAAACAGACATAGAAAA CCG and TGCCTGGACCTCCCACTGCT; –1788~–1648: TGAAGCCATAGGATGAAGATGAA AA and GCCATCAAATTGTCTGCATAAGTCA; –1545~–1375: ACCTGATACGGTATAAGGGG AAAAG and ACATGCTATGTATCCACATCATTCC; –1364~–1152: GGTTTTGTAAGGAGAA AGAAAGAGA and CATACAAAGGTGCCACACTTAAAGC; –817~–627: AGGAGTGGAAAA TGAGGAAGGGTAG and TGTAAAATGGGAACAGTCACAGGAT; –548~–449: AGAGCCACT TGTCTGCTACTTGAAA and TGGTATGGAAGCCTAGATCTGTGTA.

### RNA-Seq and analysis

Total RNA was isolated from 4 × 10^7^ MCF-7 cells treated with DMSO or 50 nM LBH589 for 12 h. RNA-Seq was carried out on the Illumina HiSeq platform. Mapping of RNA-Seq reads was performed with HISAT2 (http://ccb.jhu.edu/software/hisat2/index.shtml/) and the human RefSeq gene model (GRCh38.p10) and RSEM (http://deweylab.github.io/RSEM/) software were used to quantify gene expression, isoform-level expression. The differential analyses were analyzed with DESeq2, GO enrichment analysis was performed with GO Atools(https://pypi.org/project/goatools/).

### Luciferase reporter assays

Four different parts of the NEDD9 gene promoter regions were amplified from MDA-MB-231 cells using genomic DNA as template. The acquired sequences were then digested with MluI and XhoI and ligated into pGL3-Basic vector (#E1751, Promega, Wisconsin, US). For transcriptional activity assay, MCF-7 cells were seeded and transfected with the pGL3-*nedd9*-luc promoter (-2178/-1540/-1190/-666 to +197) reporter plasmids and PRL-TK (Renilla luciferase) using Lipofectamine 2000 (#11668019, Invitrogen). After 24 h, firefly renilla luciferase activities were determined by the dual-luciferase reporter assay (#E1910, Promega). Primers used as follow:

pGL3-*nedd9*-luc (−2178): CGACGCGTAGTAGGAAAACAGACATAGAAAACC;

pGL3-*nedd9*-luc (−1540): CGACGCGTGAGGAAAGAAGAATAAAAGCATACC;

pGL3-*nedd9*-luc (−1190): CGACGCGTCTGATGGGTGGGACCAAGGG;

pGL3-*nedd9*-luc (−666): CGACGCGTGATCTAGGCTTCCATACCACATTGT;

pGL3-*nedd9*-luc (+197): CCGCTCGAGGGAACCGCTGGTGGATCATCTTGCC.

### Statistical analysis

The statistical difference between indicated groups were analyzed by two-tailed Student’s *t*-test. The differential gene expression was analyzed by Wald test between control and treatment groups. GO enrichment analysis depended on the fisher‘s precise test. Differences were considered to be statistically significant at *p* < 0.05.

## Supplementary information

Supplementary Materials-R2-clean

## Data Availability

All sequencing raw data (GSA for Human: HRA001559) reported here are available in National Genomics Data Center (Nucleic Acids Res 2021), China National Center for Bioinformation/Beijing Institute of Genomics, Chinese Academy of Sciences by visiting https://ngdc.cncb.ac.cn/gsa.
