# Supplementary material for: Histone deacetylase inhibitors promote breast cancer metastasis by elevating NEDD9 expression
Source: Signal Transduct Target Ther. 2023 Jan 6;8:11. doi: 10.1038/s41392-022-01221-6 (PMC9816171; doi:10.1038/s41392-022-01221-6)
Supplement: Supplementary file 1 — Supplementary Materials-R2-clean [file 41392_2022_1221_MOESM1_ESM.docx]

**Supplementary Materials for**

**Histone deacetylase inhibitors promote breast cancer metastasis by elevating NEDD9 expression**

Zonglong Hu^1, 2^, Fan Wei^1^, Yi Su^1^, Yafang Wang^1^, Yanyan Shen^1^, Yanfen Fang^1^, Jian Ding^1, 2, 3*^, Yi Chen^1, 2*^

*Corresponding author. Email: [ychen@simm.ac.cn](mailto:ychen@simm.ac.cn), jding@simm.ac.cn.

**This file includes:**

Supplementary Figures 1-14 with their legends

Supplementary Tables 1-3

**
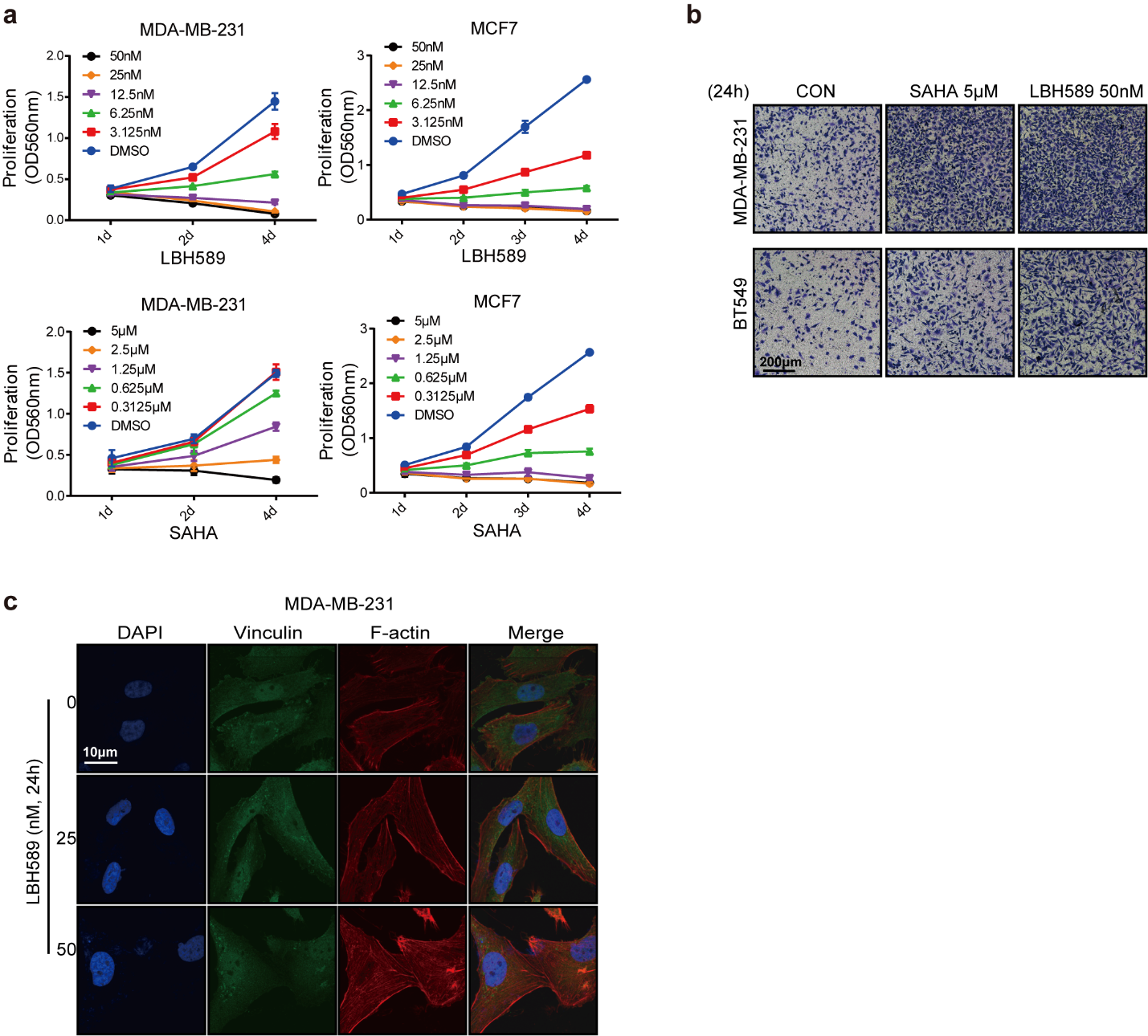
**

**Supplementary Fig. 1** Pan-HDAC inhibitors induce migration of breast cancer cells *in vitro* (Related to Fig. 1). **a** Breast cancer cells treated with HDAC inhibitors LBH589 and SAHA, and the inhibitory effects on cell proliferation were measured using the SRB assay. **b** HDAC inhibitors enhanced breast cancer cell invasiveness. Cells were treated with LBH589 or SAHA for 24 h at indicated doses, and were plated in 5% Matrigel embedded transwell chambers for 12 h to evaluate the cell motility. Scale bar, 200 μm. **c** Immunofluorescence staining analysis of focal adhesion site number characterized by vinculin staining and actin-containing invadopodia in MDA-MB-231 cells treated with HDAC inhibitors. Scale bar, 10 μm. Error bars indicate mean ± SD.


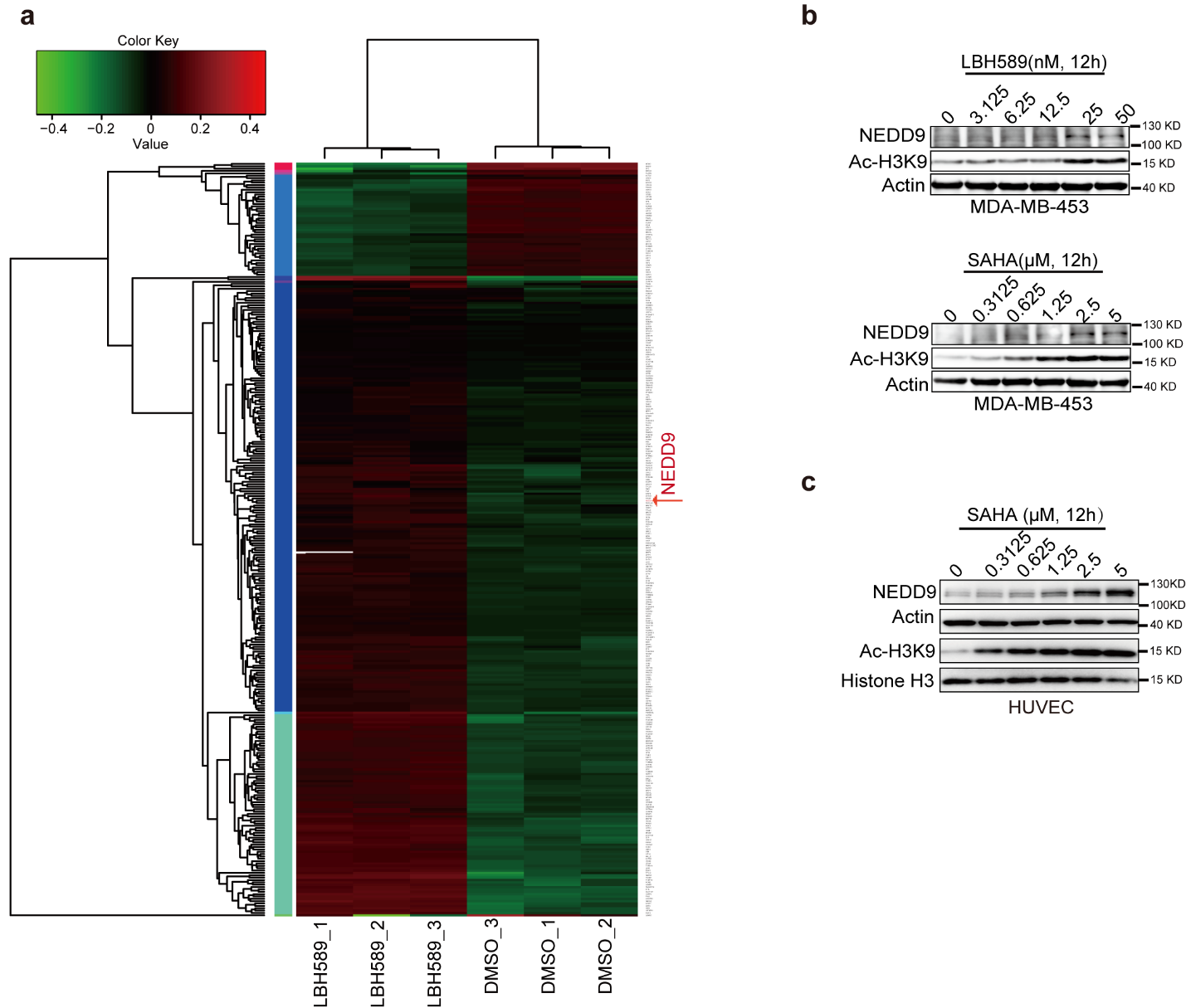


**Supplementary Fig. 2** HDAC inhibitors upregulate expression of NEDD9 in breast cancer cells (Related to Fig. 2). **a** Heatmaps of gene-expression data from RNA-Seq analysis. MCF-7 cells were treated with 50 nM LBH589 in triplicate for 12 h and the total RNA was extracted, sequenced and analyzed. Gene-expression alteration in the treated group was normalized compared with control group treated with DMSO. Red, induced (2.5-fold change); green, repressed (0.4-fold change). **b** Immunoblotting of NEDD9 expression in MDA-MB-453 cells after treatment with HDAC inhibitors. **c** Immunoblotting of NEDD9 expression in endothelial HUVEC cells after treatment with SAHA.


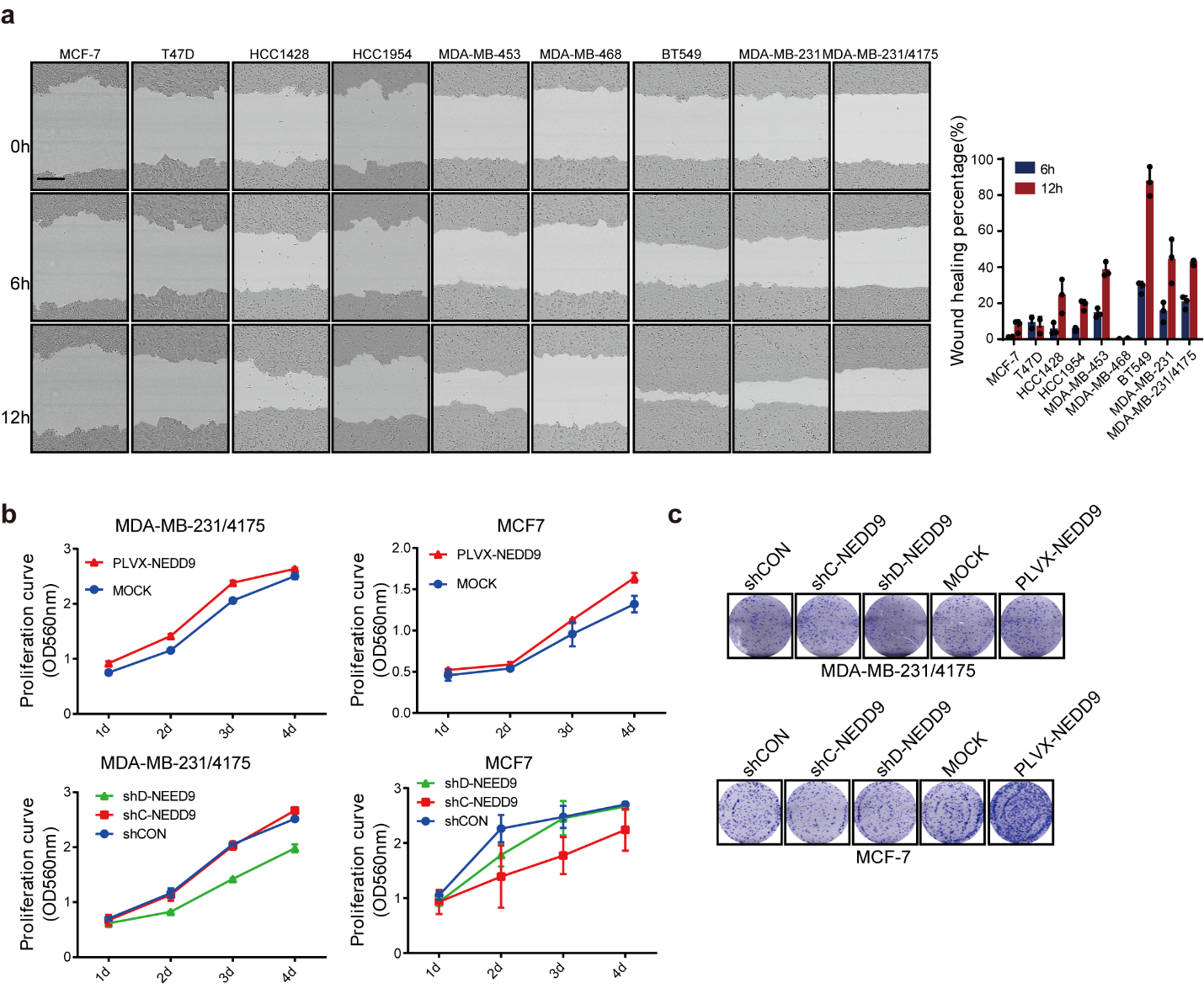


**Supplementary Fig. 3** NEDD9 promotes breast cancer metastasis (Related to Fig. 3). **a** Wound scratch assay of different breast cancer cells. Scale bar, 300 μm. SRB assay **(b)** and plate clone formation assay **(c)** showed that overexpression or knockdown of NEDD9 almost had no effects on MCF-7 and MDA-MB-231/4175 cells proliferation. Error bars indicate mean ± SD.

**
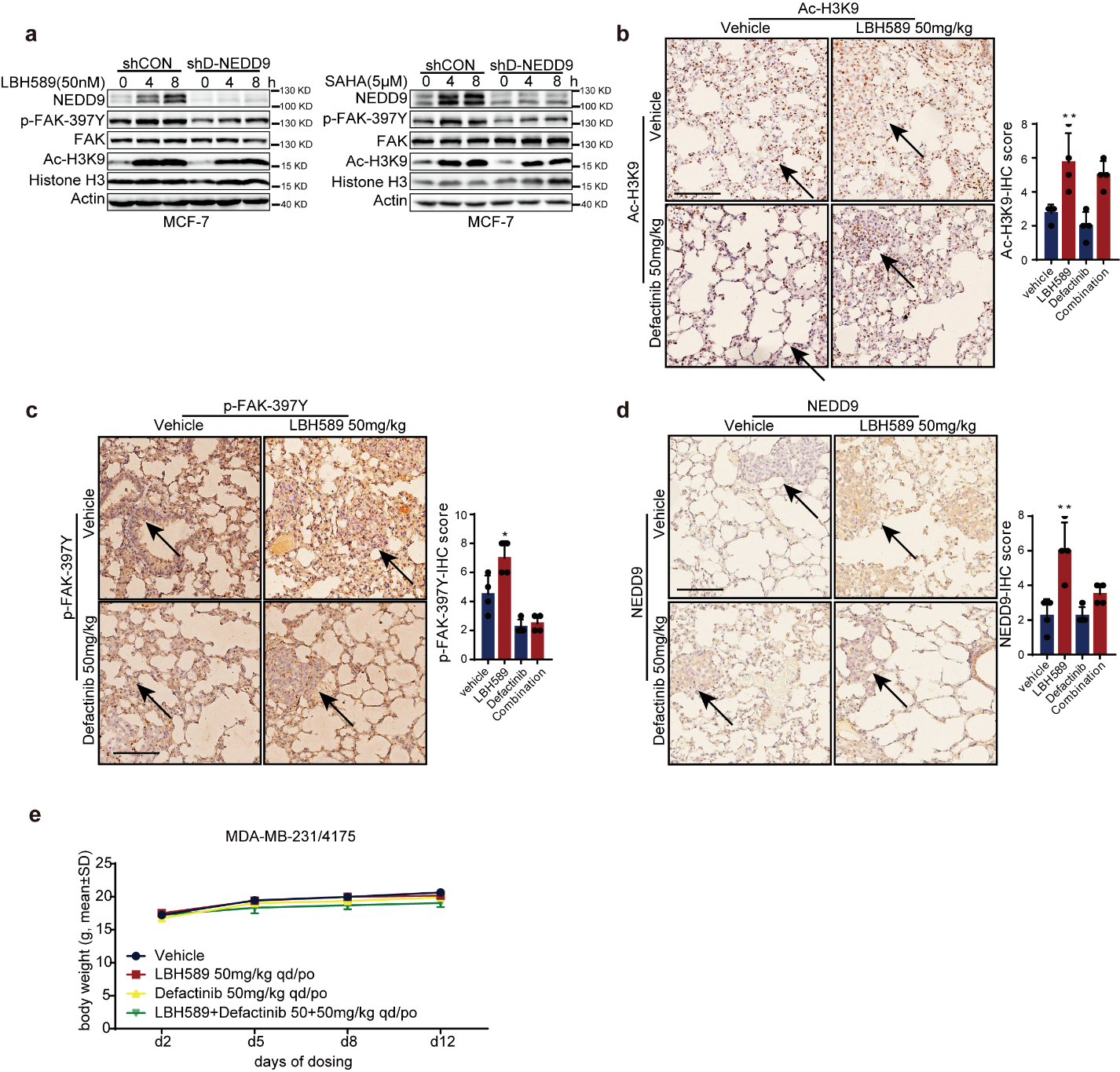
**

**Supplementary Fig. 4** Phosphorylated FAK is the potential downstream of NEDD9 regulated by HDACi (Related to Fig. 5). **a** Immunoblotting of p-FAK-397Y expression in knockdown of NEDD9 treated with HDAC inhibitors. Representative images and quantification of Ac-H3K9-**(b)**, p-FAK-397Y-**(c)** and NEDD9-stained **(d)** in lung sections. The arrows indicate positive staining. **(E)** mice body weight of combination therapy of LBH589 and defactinib. Scale bar, 100 μm. Error bars indicate mean ± SD. *p < 0.05, **p < 0.01.


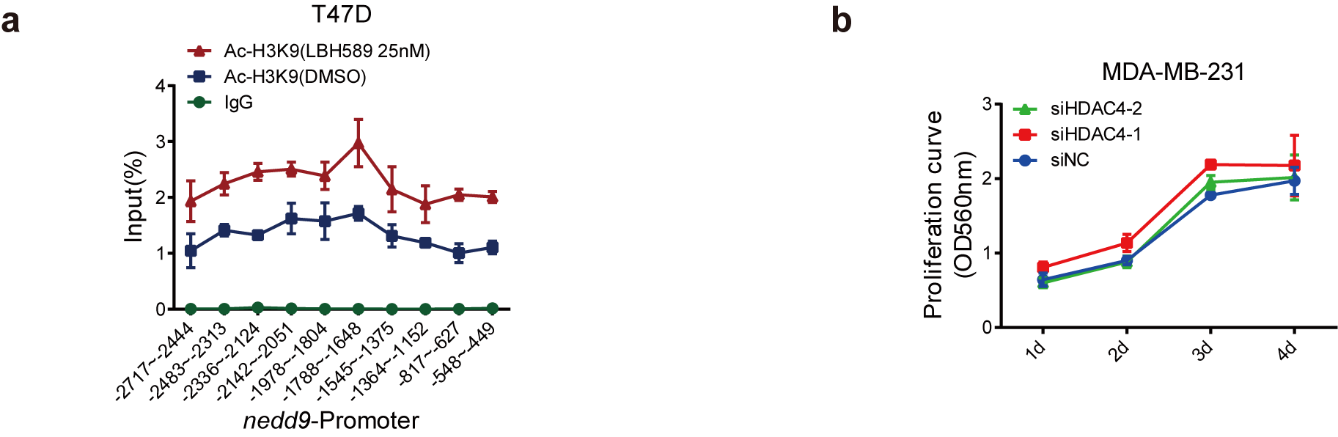


**Supplementary Fig. 5** Phosphorylated FAK is the potential downstream of NEDD9 regulated by HDACi (Related to Fig. 5). **a** ChIP immunoprecipitation of recruitment of Ac-H3K9 to the NEDD9 promoter region was observed after T47D cells treatment with HDAC inhibitors. **b** SRB assay showed that knockdown of HDAC4 had no effects on proliferation of MDA-MB-231 cells. Error bars indicate mean ± SD.

**Supplementary Table 1** A part of 1591 significantly changed genes in RNA-seq analysis (related to Fig. 2).

| Gene_name | FC(LBH589/DMSO) | Log2FC | Padjust | Significant | Regulate |
| --- | --- | --- | --- | --- | --- |
| NEDD9 | 2.898 | 1.535 | 5.49E-44 | yes | up |
| MYOD1 | 3.837 | 1.94 | 2.54E-05 | yes | up |
| WDR76 | 0.188 | -2.414 | 1.42E-83 | yes | down |
| ZFP2 | 2.789 | 1.48 | 0.000122 | yes | up |
| CCNE2 | 0.193 | -2.372 | 4.94E-78 | yes | down |
| DSCC1 | 0.233 | -2.099 | 9.21E-93 | yes | down |
| C20orf166-AS1 | 10.689 | 3.418 | 1.36E-16 | yes | up |
| ESPL1 | 0.335 | -1.579 | 6.45E-66 | yes | down |
| SEMA6A | 2.772 | 1.471 | 3.39E-33 | yes | up |
| AC005696.4 | 5.049 | 2.336 | 3.59E-13 | yes | up |
| AL023802.1 | 3.394 | 1.763 | 1.59E-05 | yes | up |
| LINC00652 | 3.061 | 1.614 | 0.000491 | yes | up |
| KCNE4 | 0.24 | -2.057 | 3.62E-89 | yes | down |
| LINC01419 | 16.134 | 4.012 | 3.25E-25 | yes | up |
| RAET1G | 5.109 | 2.353 | 2.35E-15 | yes | up |
| E2F7 | 0.222 | -2.17 | 3.87E-79 | yes | down |
| AC009303.4 | 2.878 | 1.525 | 0.000138 | yes | up |
| C17orf53 | 0.357 | -1.484 | 2.29E-17 | yes | down |
| LINC00663 | 2.898 | 1.535 | 4.49E-06 | yes | up |
| SDAD1P1 | 4.701 | 2.233 | 6.62E-27 | yes | up |
| MMP1 | 3.48 | 1.799 | 0.000102 | yes | up |
| RBM11 | 3.883 | 1.957 | 1.09E-06 | yes | up |
| PLXNA2 | 5.548 | 2.472 | 1.6E-19 | yes | up |
| HAPLN3 | 3.983 | 1.994 | 2.84E-11 | yes | up |
| MUC12 | 3.392 | 1.762 | 0.000168 | yes | up |
| ACTN3 | 9.063 | 3.18 | 3.66E-39 | yes | up |
| FBXO5 | 0.223 | -2.168 | 3.94E-79 | yes | down |
| CAMKV | 3.087 | 1.626 | 5.35E-07 | yes | up |
| SAMD12-AS1 | 3.568 | 1.835 | 6.36E-06 | yes | up |
| LRRN1 | 5.028 | 2.33 | 3.07E-07 | yes | up |

**Supplementary Table 2** Top 20 GO were enriched by analyzing 1591 significantly changed genes (related to Fig. 2).

| GO ID | Term Type | Description | Pvalue_uncorrected |
| --- | --- | --- | --- |
| GO:0006270 | BP | DNA replication initiation | 1.04581E-11 |
| GO:0000722 | BP | telomere maintenance via recombination | 4.27728E-11 |
| GO:0006310 | BP | DNA recombination | 5.33473E-11 |
| GO:0006312 | BP | mitotic recombination | 6.7247E-11 |
| GO:0006260 | BP | DNA replication | 9.83686E-11 |
| GO:0050911 | BP | detection of chemical stimulus involved in sensory perception of smell | 1.21509E-10 |
| GO:0050907 | BP | detection of chemical stimulus involved in sensory perception | 1.61465E-10 |
| GO:0007049 | BP | cell cycle | 1.70805E-10 |
| GO:0051276 | BP | chromosome organization | 1.81183E-10 |
| GO:0009593 | BP | detection of chemical stimulus | 2.40467E-10 |
| GO:0022610 | BP | biological adhesion | 6.05469E-07 |
| GO:0005856 | CC | cytoskeleton | 1.90179E-07 |
| GO:0098797 | CC | plasma membrane protein complex | 5.5878E-07 |
| GO:0030054 | CC | cell junction | 1.08945E-06 |
| GO:0004984 | MF | olfactory receptor activity | 1.21509E-10 |
| GO:0003723 | MF | RNA binding | 3.5803E-10 |
| GO:0005509 | MF | calcium ion binding | 1.62167E-09 |
| GO:0008094 | MF | DNA-dependent ATPase activity | 1.80664E-07 |
| GO:0043142 | MF | single-stranded DNA-dependent ATPase activity | 4.34856E-07 |
| GO:0003678 | MF | DNA helicase activity | 6.92901E-07 |

**Supplementary Table 3** Seven significantly changed genes in venn analysis (related to Fig. 2).

| Gene_name | FC(LBH589/DMSO) | Log2FC | Pvalue | Padjust | Significant | Regulate |
| --- | --- | --- | --- | --- | --- | --- |
| SORBS1 | 4.503 | 2.171 | 1.4E-32 | 3E-31 | yes | up |
| HCK | 6.338 | 2.664 | 5.33E-20 | 6.03E-19 | yes | up |
| MSN | 4.272 | 2.095 | 1.3E-10 | 7.61E-10 | yes | up |
| S100A9 | 2.619 | 1.389 | 7.04E-19 | 7.45E-18 | yes | up |
| NPHP1 | 3.874 | 1.954 | 7.28E-17 | 6.83E-16 | yes | up |
| NEDD9 | 2.898 | 1.535 | 1.64E-45 | 5.49E-44 | yes | up |
| SORBS2 | 2.896 | 1.534 | 0.000164 | 0.000505 | yes | up |


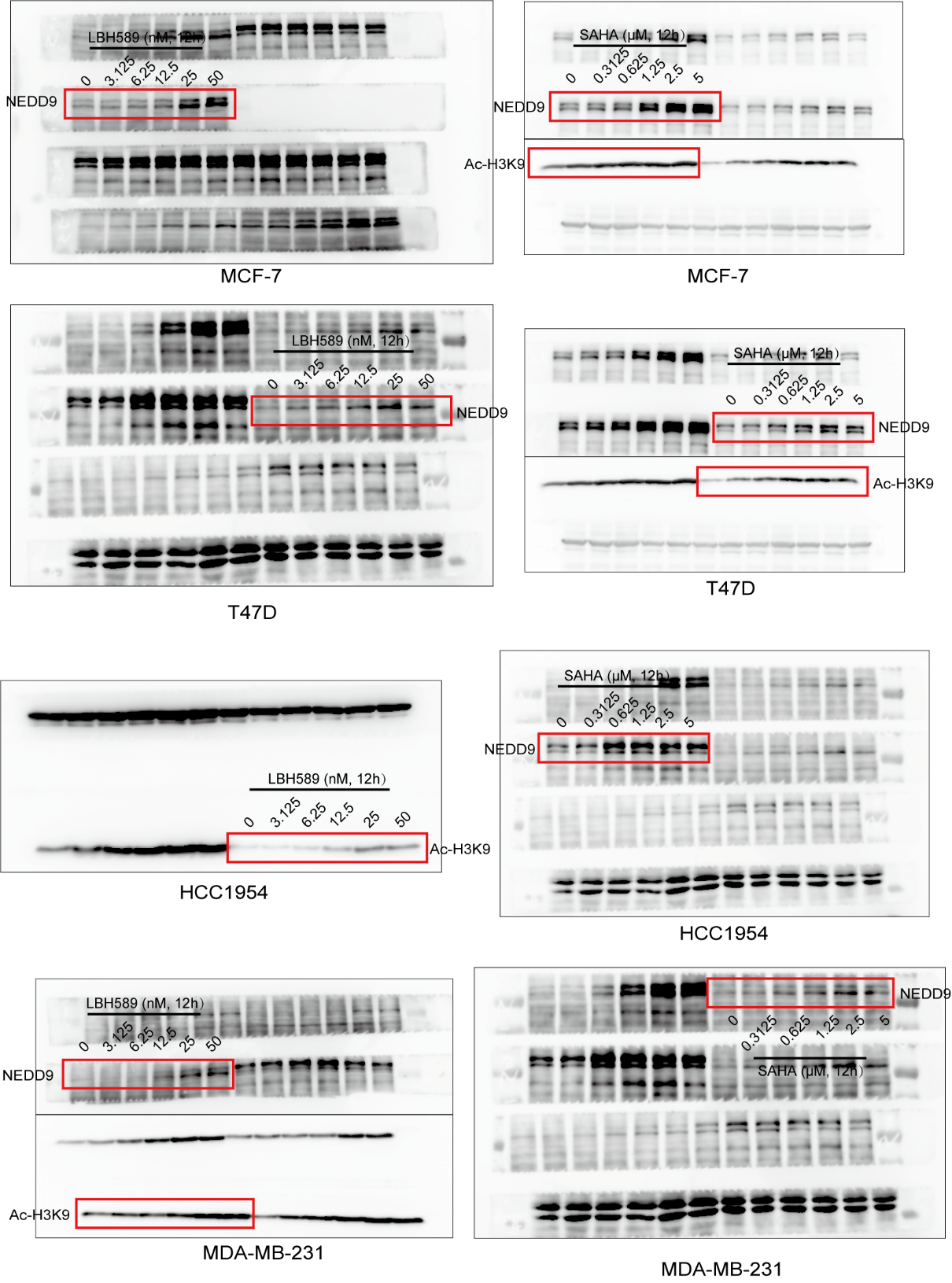


**Supplementary Fig. 6** Raw data of western blot related to Fig. 2e.


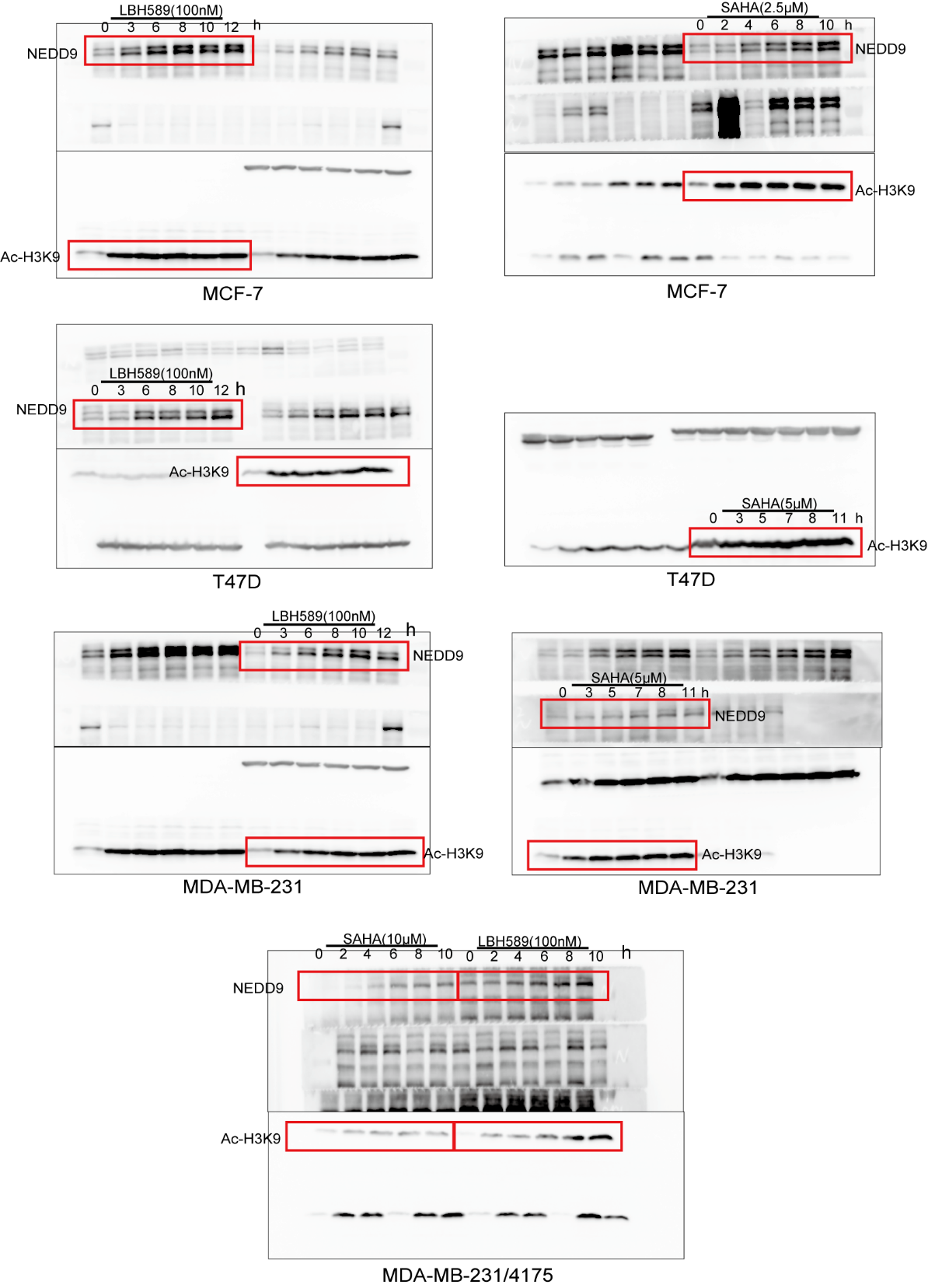


**Supplementary Fig. 7** Raw data of western blot related to Fig. 2f.


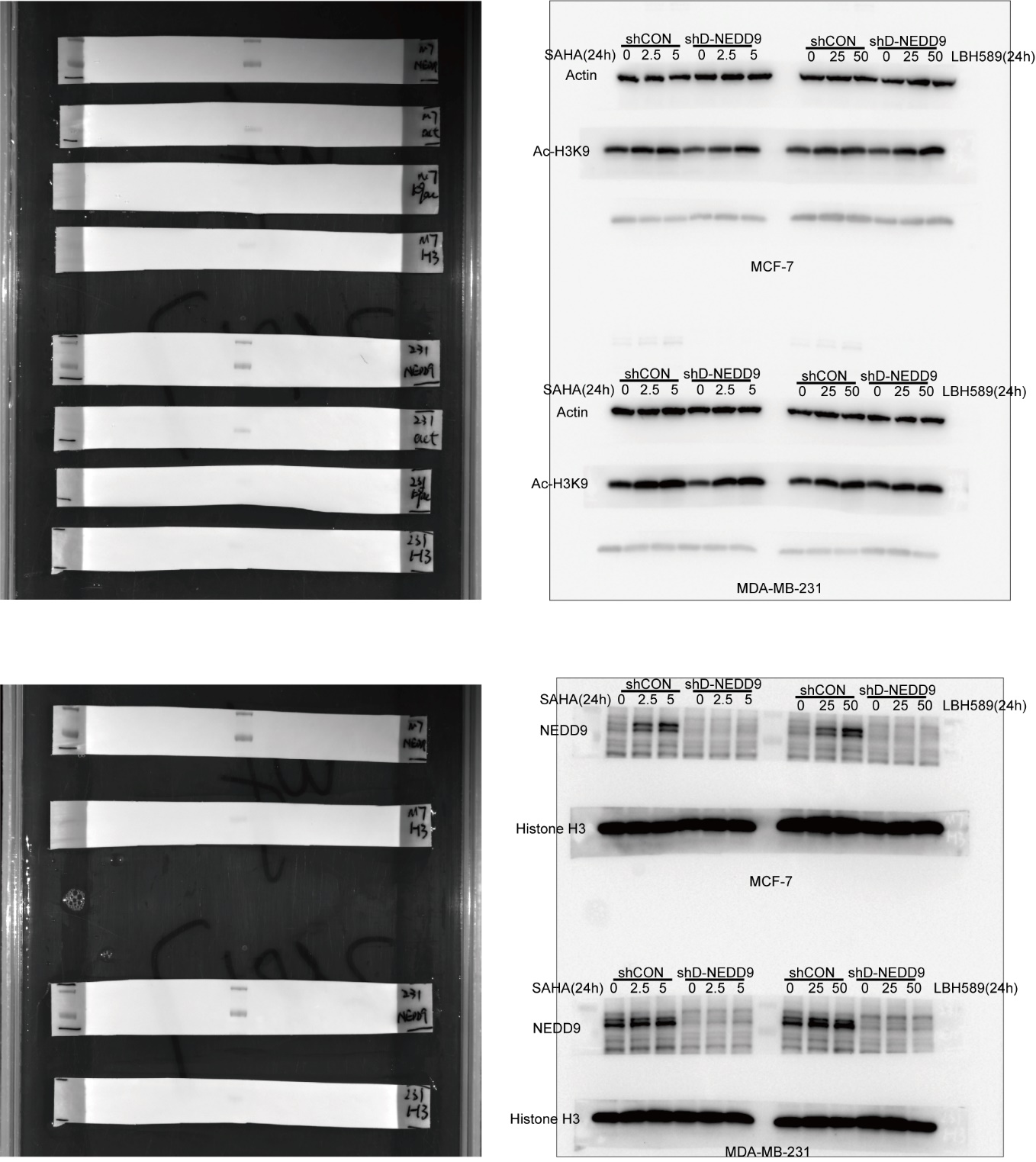


**Supplementary Fig. 8** Raw data of western blot related to Fig. 4c.


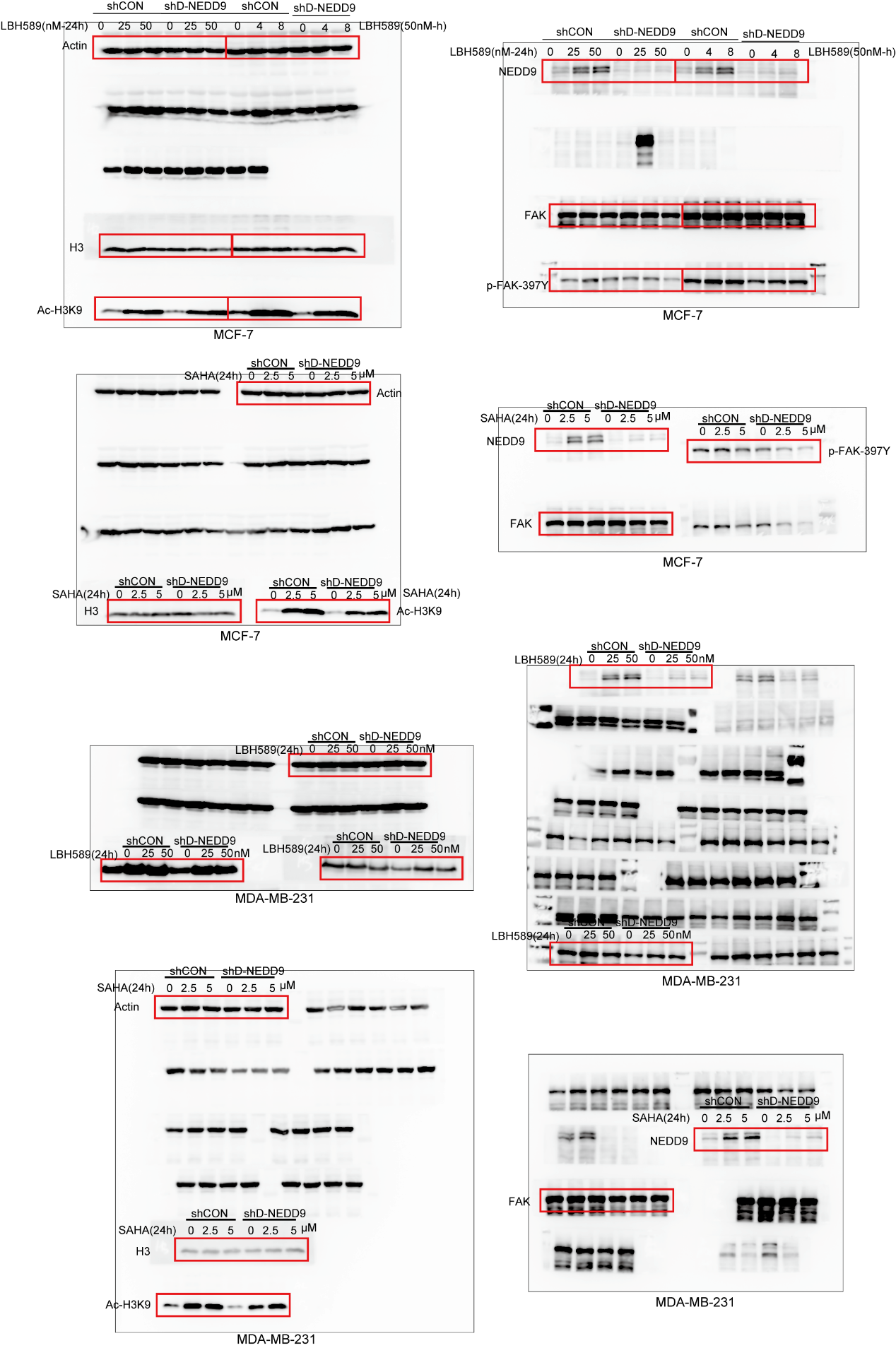


**Supplementary Fig. 9** Raw data of western blot related to Fig. 5e.


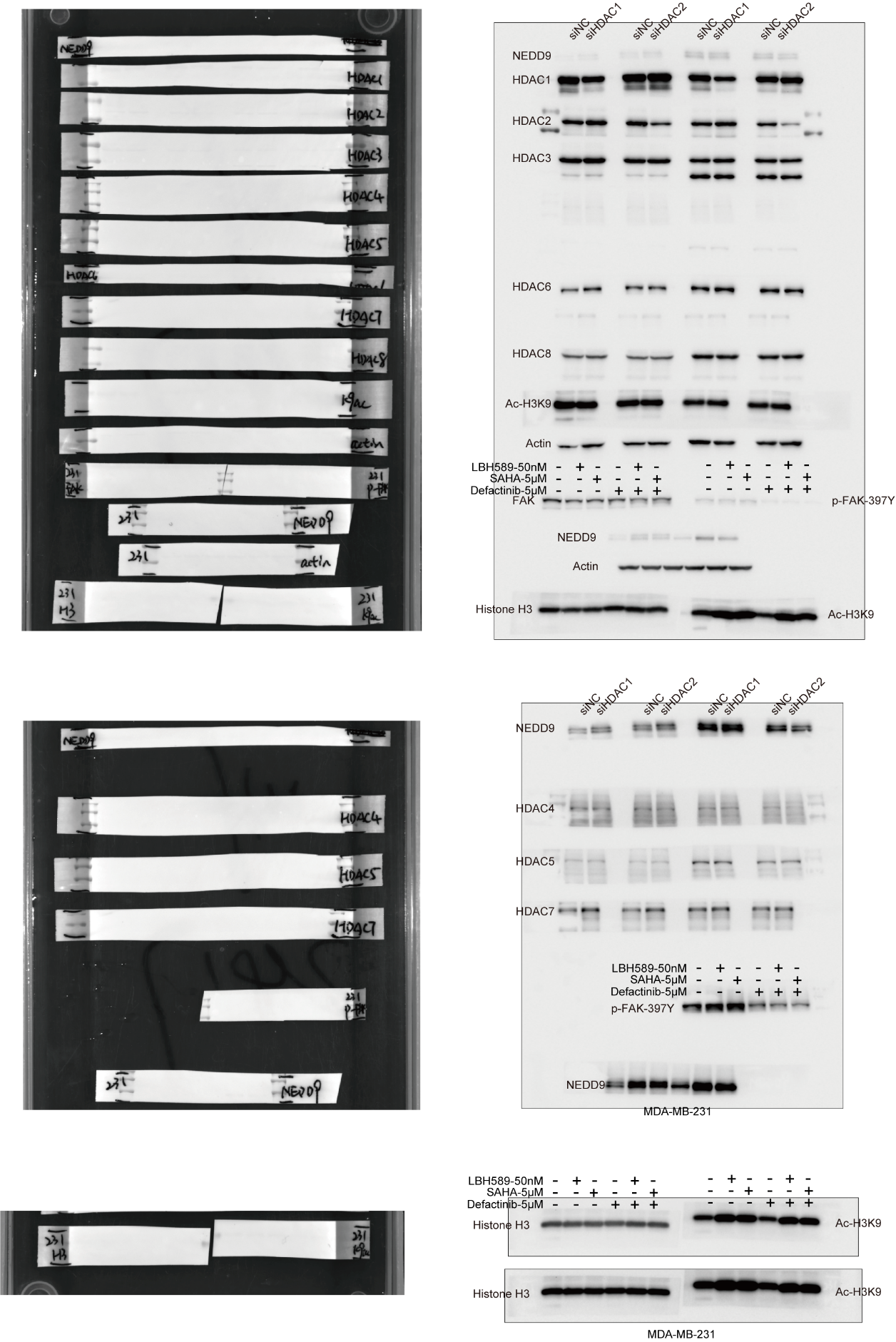


**Supplementary Fig. 10** Raw data of western blot related to Fig. 5g and Fig 6b.


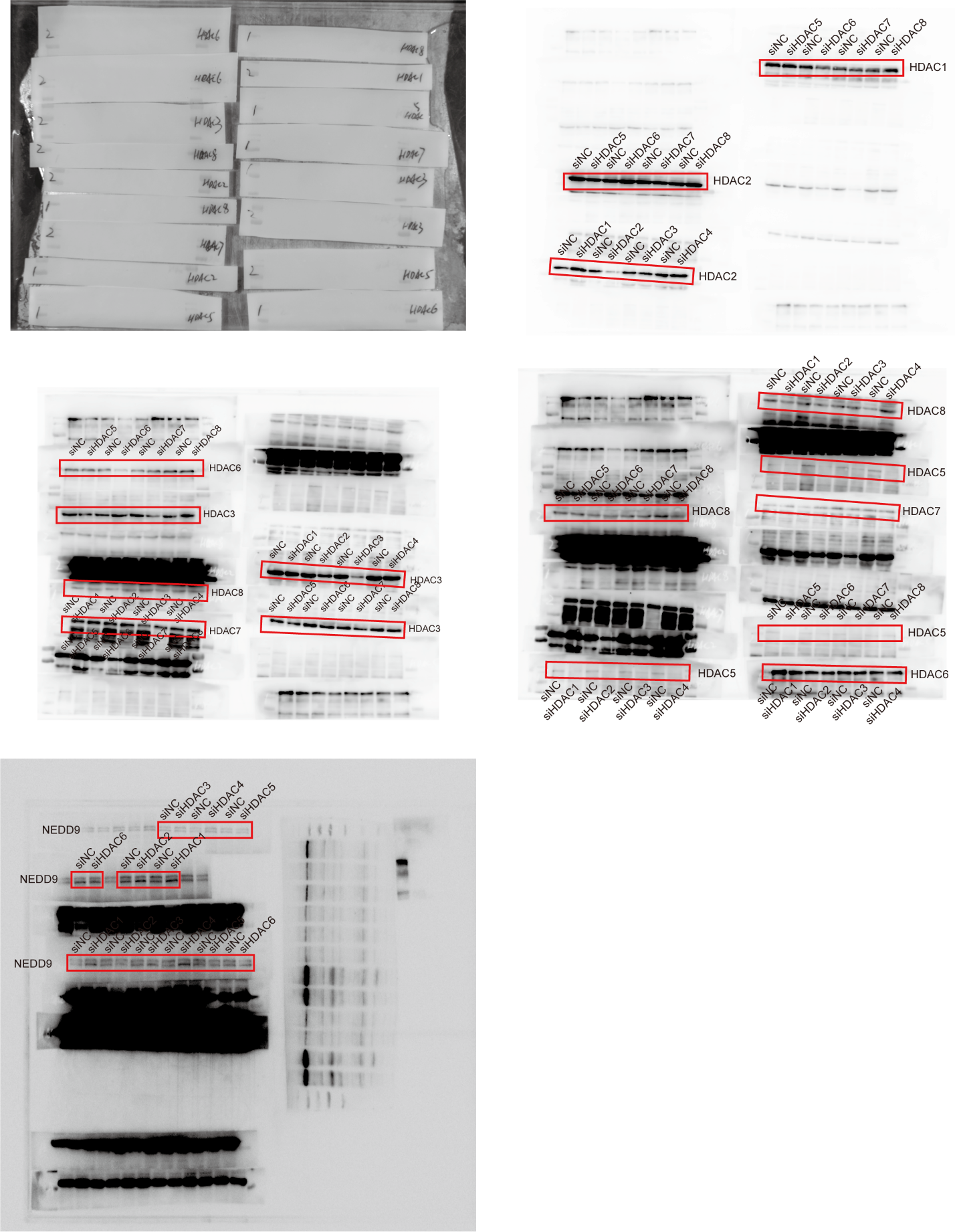


**Supplementary Fig. 11** Raw data of western blot related to Fig. 6b.


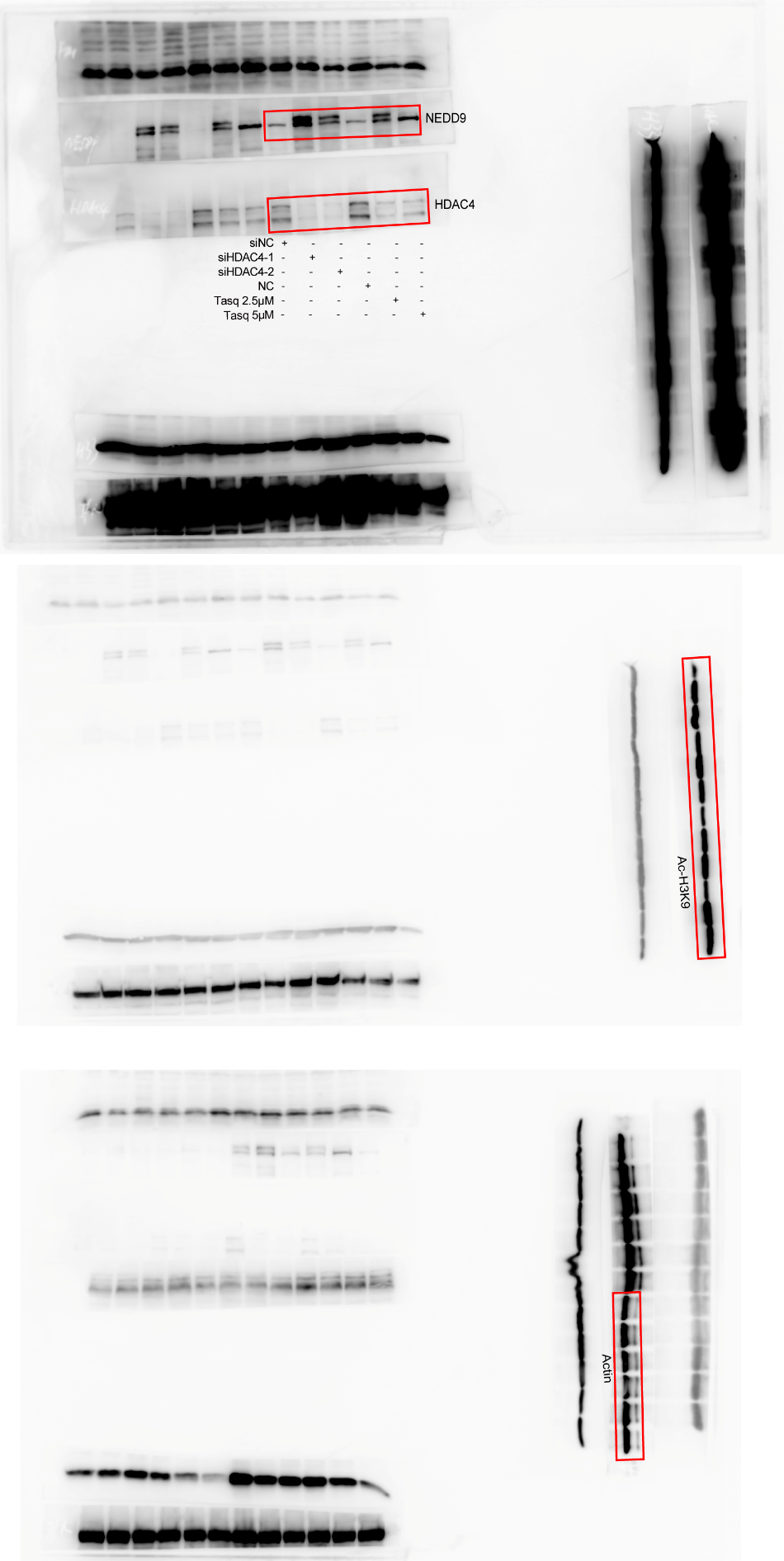


**Supplementary Fig. 12** Raw data of western blot related to Fig. 6e.


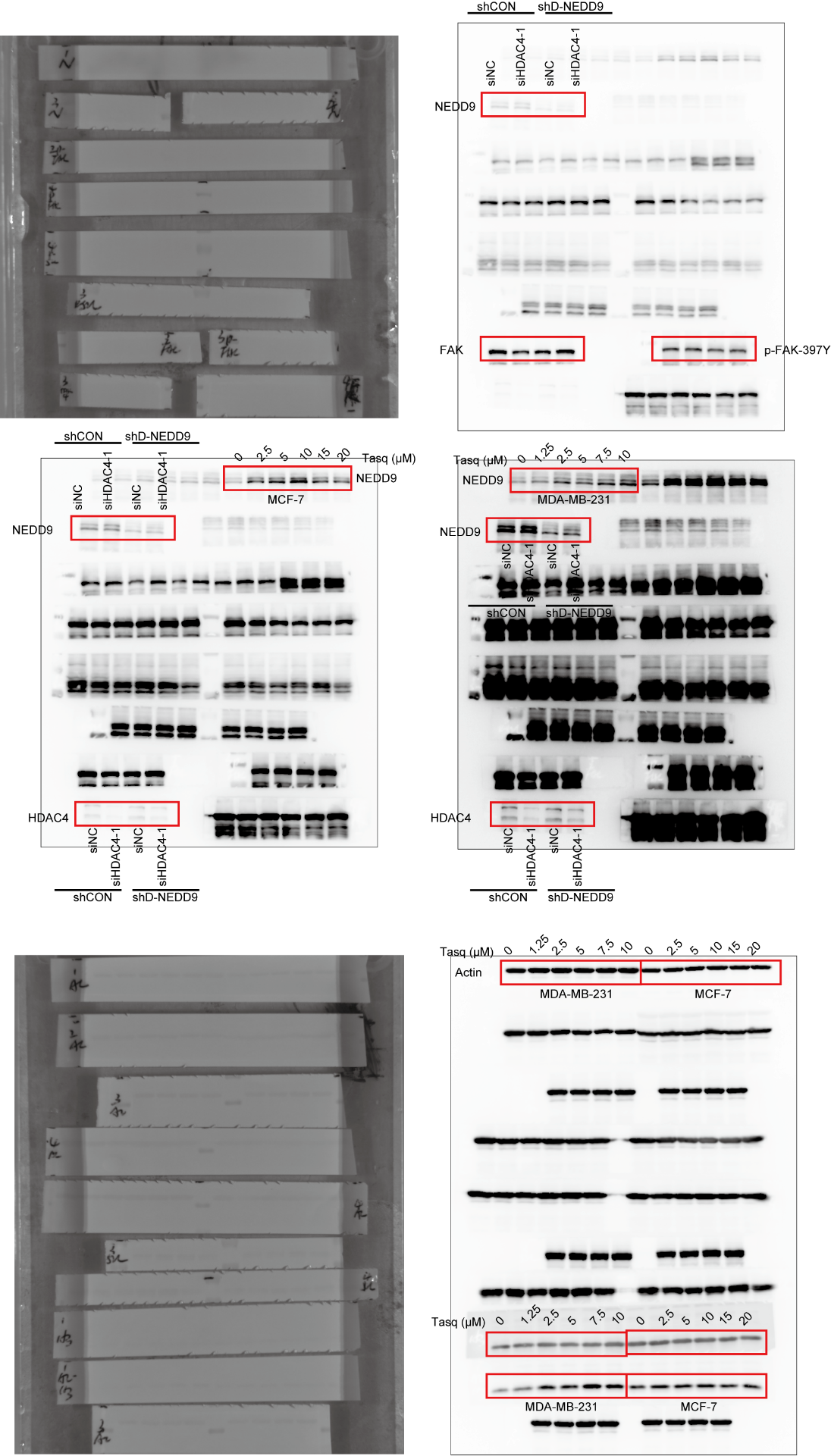


**Supplementary Fig. 13** Raw data of western blot related to Fig. 6g and Fig. 6b.


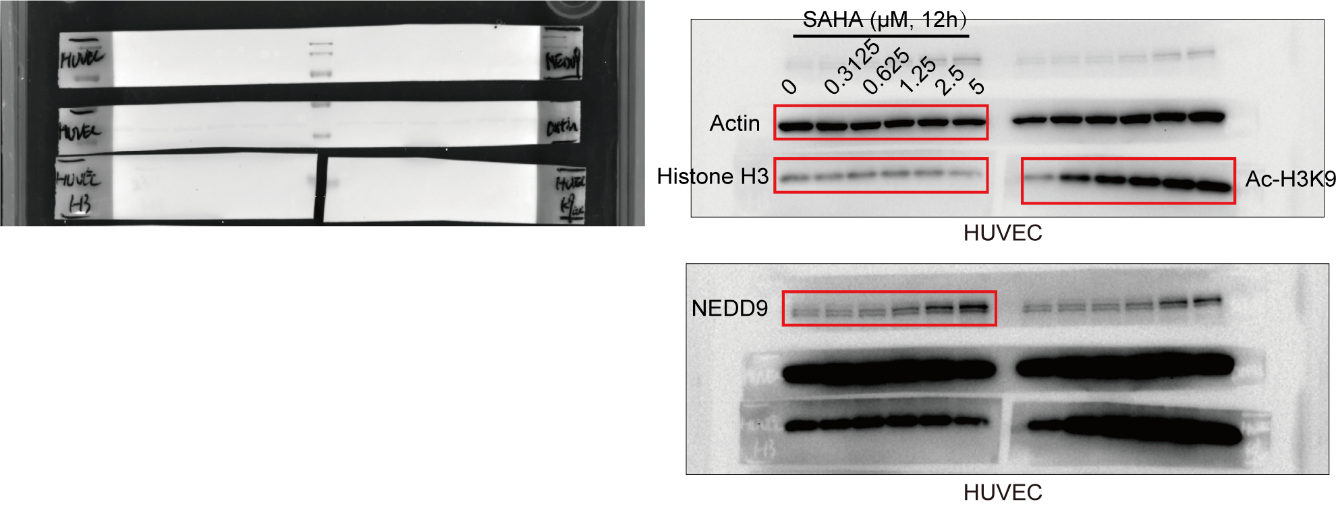


**Supplementary Fig. 14** Raw data of western blot related to Supplementary Fig. 2c.
